# Activation of Small Molecules by Heavier Analogs of Cyclic(Alkyl)(Amino)Carbenes (CAACs): A DFT Study

**DOI:** 10.1021/acs.inorgchem.6c01118

**Published:** 2026-07-10

**Authors:** Britney Cai, Petra Vasko

**Affiliations:** † Department of Chemistry, 3835 University of Helsinki, P.O. Box 55 (A. I. Virtasen Aukio 1), Helsinki 00014, Finland; ‡ Department of Chemistry, 2129 University of Calgary, 2500 University Dr NW, Calgary, Alberta T2N 1N4, Canada

## Abstract

Cyclic (alkyl)­(amino)­carbenes (CAACs), a subclass of N-heterocyclic carbenes (NHCs), are powerful tools in coordination chemistry due to their strong σ-donor and π-acceptor abilities. The heavier analogs of carbenes CAAEs (E = Si–Pb), however, are not as ubiquitous but may have potential in small-molecule activation and catalysis. Here, quantum chemical computations using density functional theory (DFT) were utilized to investigate the analogs of CAACs (CAAE, E = C–Pb), in which the compounds were assessed to determine the electronic properties and reactivity toward small molecules H_2_, NH_3_, and CO_2_. The activation energies for small-molecule bond cleavage were found to increase as the central element E became heavier, which may be attributed to changes in the Lewis basicity. In addition, the differences in NH_3_ oxidative addition, Werner coordination complex-type adduct formation, and proton-shuttling mechanisms were investigated. Carbon dioxide could not be activated by the heaviest germylene, stannylene, or plumbylene derivatives, but the CAAC and CAASi species interacted with CO_2_ via different coordination modes. The results were comparable to those of experimentally assessed systems, indicating the feasibility of synthesizing and isolating these heavier CAAC derivatives and their ability to activate small molecules.

## Graphical abstract

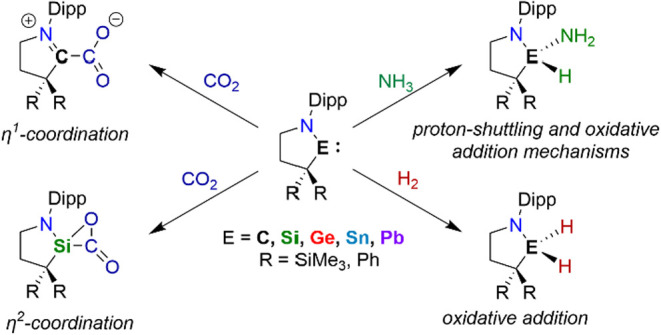

## Introduction

Cyclic (alkyl)­(amino)­carbenes (CAACs) are widely used as neutral ligands in organometallic and coordination chemistry due to their easily tunable nature.^1^ However, CAACs are reactive on their own accord, and as such, these lightest group 14 congeners have been well studied and utilized in various applications, for example, in the metal-free activation of small molecules such as H_2_, NH_3_, CO_2_, and other less polar bonds.^2–5^ Recently, interest in the chemistry of heavier analogs, especially CAASi and CAAGe, has been growing, and several studies have been conducted on the reactivity of these species toward various small molecules.^6,7^ The heavier carbene analogs CAAEs (E = Si, Ge, Sn, Pb) feature a group 14 element in a divalent state with a lone electron pair as the highest occupied molecular orbital (HOMO) and a vacant p-orbital as the lowest unoccupied molecular orbital (LUMO) (Figure 1a). These frontier molecular orbital (FMO) characteristics allow for ambiphilic reactivity behavior for CAAEs, which can be exploited, for example, in small-molecule activation and catalysis akin to transition metal complexes.^8,9^ Importantly, the singlet ground state of the CAAE analogs is increasingly stabilized upon descending the group due to the increasing radial extension of the ns and np orbitals as the atomic number increases.^10^ This, in turn, directly affects the ligating, i.e., σ-donating and π-accepting, and reactivity properties of the heavier CAAEs. Commonly, the CAAE heterocyclic backbone (saturated vs unsaturated) and substituents, such as steric and electronic properties, can be easily modified. Overall, ligand design and a fundamental understanding of the electronic properties of CAAEs are crucial for constructing novel reactive systems with potential applications, such as catalysis.

**Figure 1:**
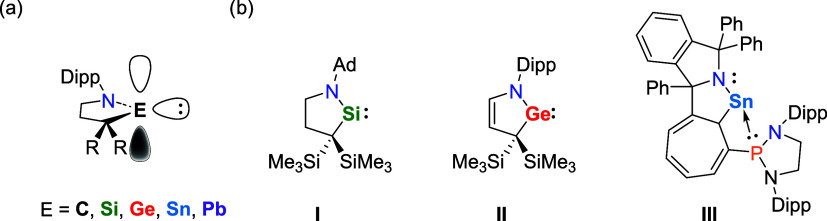
(a) Frontier molecular orbitals of CAAE (E = C, Si, Ge, Sn, Pb), and (b) synthetically attainable CAAEs **I–III** relevant to this work.

Although CAACs have been studied extensively,^3,11,12^ the chemistry of heavier congeners has slowly started to gain traction. Earlier, the availability of appropriate precursors and synthetic procedures hindered the progress in this field. Nevertheless, the synthesis, characterization, and reactivity studies of cyclic (alkyl)­(amino)­silylene **I** (Figure 1b) were reported in 2016.^6^ CAASi **I** was found to be a better electron donor than N-heterocyclic silylenes (NHSi), and it readily underwent cycloadditions with alkynes and insertion into the Si–H bond of Et_3_SiH. The germanium analog **II** was reported in the same year and was characterized by the enhanced electrophilicity (i.e., stabilized LUMO) of the Ge center and higher energy lone pair orbital compared to NHGe.^7^ Stannylene **III** was synthesized serendipitously via a transient stannyne.^13^ The stannyne underwent a sequence of Büchner ring expansions and sigmatropic shifts to finally yield the thermodynamic product **III**. In contrast to the previous examples **I** and **II**, CAASn **III** exhibits a pyramidal N atom adjacent to the group 14 element. The pyramidal geometry of nitrogen is a sign of a highly localized lone electron pair, whereas the trigonal planar nitrogen atoms in **I** and **II** indicate the delocalization of electron density toward the element E. To date, no CAAPb derivatives have been isolated.

Given the widespread use and activity of CAACs, it is of high interest and importance to study heavier analogs, their ligand properties, and reactivity. In this respect, the Phukan group recently reported theoretical studies on the electronic structures of five-membered CAASi and CAAGe species and six-membered cyclic (alkyl)­(amino)­silylenes (CAASi) and their reactivity toward a variety of small molecules.^14,15^ Their investigations probed the potential of the proposed CAASis and CAAGes for the metal-free activation of small molecules and catalysis. The studied silylenes and germylenes had wider singlet–triplet gaps compared to their carbene congeners, suggesting them to be stable enough for experimental isolation. However, some of the activation barriers to cleave the strong small-molecule bonds in order to achieve formal oxidative addition proved to be high and not feasible to realize in an experimental setting.

Herein, we investigated the electronic properties of the heavier group 14 analogs of conventional CAACs (i.e., CAAEs, E = Si, Ge, Sn, Pb) by density functional theory (DFT) methods (Figure 2). In addition, we broadened the study to investigate the compounds’ ability to activate small molecules, such as H_2_, NH_3_, and CO_2_. The observed trends and predicted reactivities are discussed in detail below.

**Figure 2:**
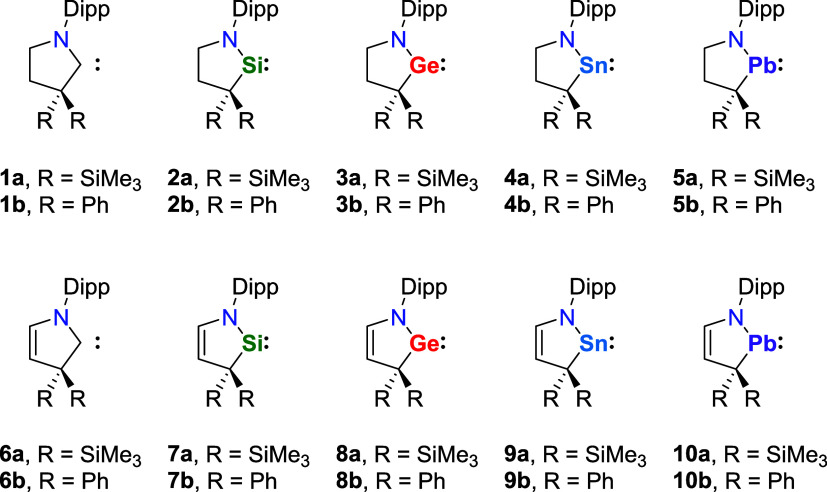
Schematic representation of CAAE (E = C–Pb) frameworks considered in the study.

## Computational Details

All density functional theory-based geometry optimization calculations were performed using the Gaussian16 (Rev.C.02) program,^16^ with the PBE0 hybrid exchange–correlation functional^17–19^ in conjunction with the Def2-SVP^20,21^ basis set and Grimme’s empirical dispersion correction with Becke–Johnson damping (D3­(BJ)).^22^ In addition, single-point calculations were performed on the optimized geometries with the same functional combined with the Def2-TZVP basis set and solvent (toluene) by using the polarizable continuum model (PCM). For Sn and Pb, effective core potentials (ECP) were used to model the core electrons. An ultrafine integration grid was used in all calculations. Optimization and frequency calculations were performed at a lower level of theory to identify the nature of the stationary points. The ground-state geometries exhibited real vibrational frequencies only, while the transition state (TS) geometries were characterized as saddle points in the presence of one imaginary frequency. NBO calculations were conducted at the PBE0-D3­(BJ)/Def2-TZVP level of theory with the NBO3.1 program, as implemented in Gaussian16. EDA-NOCV^23–26^ calculations were performed using ADF 2022.103, as implemented in the Amsterdam Modeling Suite 2025.105 (AMS).^27^ The calculations used single-point geometries at the ZORA-PBE0-D3­(BJ)/TZ2P level of theory, with no frozen core and numerical quality keyword set as “good”. The reported energies correspond to the Def2-TZVP single-point energies corrected with the Def2-SVP calculated Gibbs free energy correction term at 298 K.

## Results and Discussion

### Geometry Optimization and Electronic Structures

The structures of group 14 cyclic tetrylenes **1–10** were first optimized by density functional theory (DFT) at the PBE0-D3­(BJ)/Def2-SVP level. The cyclic (alkyl)­(amino)­carbene (CAAC) framework presented in Figure 2 was chosen to compare the differences between the heavier CAAC analogs and those of the well-known carbene congeners and their reactivity.^3,11,12,28^ The easily tunable nature of the CAAC backbone enabled the synthesis and characterization of numerous compounds in recent years. Herein, the study is limited to a five-membered heterocycle with the backbone being either saturated (compounds **1–5**) or unsaturated (compounds **6–10**) (Figure 2). In addition, two different R groups as substituents were considered (R = SiMe_3_ (**a**); Ph (**b**)). The sterically demanding, electron-donating trimethylsilyl (TMS) substituents next to the reactive group 14 element were chosen, first, to offer kinetic stabilization, especially by reducing the probability for dimerization through the element E, and second, to donate electron density to the heterocycle in the form of thermodynamic stabilization. The second R substituent, the phenyl group, was chosen due to its smaller size as well as different electronic properties compared to TMS. The aryl substituents were assumed to electronically stabilize CAAEs by both inductively withdrawing and simultaneously donating electron density to the system via π-electrons.^1,29^ The effect of electronic stabilization from aromaticity is also governed by the saturation of the heterocyclic backbone, but due to the conjugated π-system, the phenyl substituents can also donate electron density throughout the unsaturated CAAC framework.

The computed geometric parameters (E–C and E–N bond lengths and C–E–N angles), Mulliken charges, NBO-derived natural charges, Wiberg bond indices (WBI), and valence orbital configurations for elements E are presented in Table 1. The calculated bond parameters for carbenes **1a**,**b** and **6a**,**b** are in good agreement with the experimentally obtained values for a similar compound, ^Me^CAAC, with four methyl substituents in the unsaturated heterocycle (see further details in the SI).^30^ Similarly, the geometry of **8a** is well reproduced by our computations.^7^ In general, the saturation of the backbone or identity of substituents R has a negligible effect on the E–C and E–N bond lengths and the C–E–N angle. Increasing the tetrel element size leads to lengthening of both the E–C and E–N bond distances, as expected. An opposite trend was observed for the C–E–N bond angles, which decreased when moving down the group. This is due to the increase of the size of the element E as well as the more s-character-like lone electron pair.^31^ The ns and np valence orbital natural electron configurations obtained from NBO analyses (Table 1) indicate that s/p-orbital mixing is most pronounced in the lightest CAAC derivatives (compounds **1a**,**b** and **6a**,**b**). Moreover, the short C–N and C–C bonds in **1a**,**b** and **6a**,**b** and WBI values greater than 1 agree with these species exhibiting significant multiple bond character around the carbenic carbon, which is not observed in the heavier analogs. As such, the occupancy of the ns-orbital increases as the group descends, and for the heaviest CAAPb analogs, the lone pair is located almost exclusively on the nonbonding 6s orbital.

**Table 1:** Calculated Bond Lengths (Å), Angles (°), Mulliken (*q*_M_) and Natural Charges (q_NPA_), and Wiberg Bond Indices (WBI)

**CAAE**	**E–C**	**E–N**	**∠C–E–N**	***q***_**M**_ (E)	**q**_**NPA**_ (E)	**WBI E–N**	**WBI E–C (sp** ^**3**^ **)**	**Natural electron configuration at E (ns and np orbitals)**
**1a**	1.486	1.320	106.5	–0.544	0.117	1.42	1.12	2s(1.23)2p(2.63)
**1b**	1.534	1.310	104.9	–0.578	0.173	1.48	0.96	2s(1.30)2p(2.51)
**2a**	1.928	1.739	90.4	–0.062	1.131	0.72	0.64	3s(1.58)3p(1.26)
**2b**	1.983	1.738	88.9	0.026	1.095	0.73	0.58	3s(1.63)3p(1.25)
**3a**	2.028	1.839	87.4	0.076	1.056	0.76	0.68	4s(1.66)4p(1.27)
**3b**	2.078	1.839	86.1	0.167	1.043	0.75	0.60	4s(1.71)4p(1.23)
**4a**	2.242	2.054	81.9	0.424	1.270	0.57	0.51	5s(1.78)5p(0.94)
**4b**	2.283	2.056	80.7	0.488	1.170	0.62	0.52	5s(1.79)5p(1.03)
**5a**	2.343	2.156	79.4	0.512	1.266	0.55	0.50	6s(1.86)6p(0.87)
**5b**	2.377	2.160	78.5	0.566	1.168	0.60	0.51	6s(1.86)6p(0.97)
**6a**	1.486	1.345	103.5	–0.573	0.054	1.34	1.14	2s(1.27)2p(2.66)
**6b**	1.531	1.323	103.4	–0.640	0.173	1.41	0.96	2s(1.32)2p(2.48)
**7a**	1.921	1.776	88.3	–0.081	1.034	0.70	0.69	3s(1.59)3p(1.35)
**7b**	1.988	1.767	87.8	0.033	1.087	0.66	0.57	3s(1.65)3p(1.24)
**8a**	2.012	1.879	85.5	0.052	0.985	0.71	0.71	4s(1.68)4p(1.32)
**8b**	2.083	1.873	85.1	0.186	1.043	0.66	0.60	4s(1.73)4p(1.21)
**9a**	2.221	2.098	80.2	0.366	1.181	0.54	0.56	5s(1.78)5p(1.03)
**9b**	2.307	2.102	79.4	0.500	1.144	0.53	0.52	5s(1.81)5p(1.04)
**10a**	2.317	2.203	77.8	0.440	1.185	0.52	0.56	6s(1.86)6p(0.95)
**10b**	2.418	2.213	76.8	0.573	1.127	0.51	0.50	6s(1.88)6p(0.99)

The formal charges at element E were evaluated by calculating both the Mulliken and natural charges (Table 1). Interestingly, the NPA charges for CAAE (E = Si–Pb) are consistently close to 1, which is significantly less than the expected +2 for formally E­(II) centers. This is due to the population of the vacant p-orbital on the element E. Indeed, the NBO method characterizes the N–E bonding solely as dative bonding originating from the N-based lone pair donation to the E p-orbital, highlighting the shortcomings of this method for heavier element-containing compounds.

Inspection of the frontier molecular orbitals (FMOs) and the HOMO–LUMO gaps revealed expected trends. Considering the saturated series of analogs first, the FMOs for compounds **1a**–**5a** are mostly composed of the lone electron pair orbital on element E (HOMO) and the corresponding empty p-orbital (LUMO) (Figure 3 and SI). The heavier the element E becomes, the purer the π-type orbital of the LUMO, with less contribution from the substituents. Additionally, for CAAPb **5a**, the HOMO–2 instead of the HOMO is characterized as the lone pair orbital. Compound **1a** has the highest LUMO energy of this series, −0.37 eV, which suggests that **1a** is the weakest π-acceptor in the series. The HOMO energies for **2a**–**5a** are relatively similar to those of **1a** and decrease within ± 0.4 eV of each other (−5.76, −6.12, −6.16, −6.01, −5.88 eV for **1a**–**5a**, respectively). In comparison, the LUMO energies for **2a**–**5a** have a larger relative difference to **1a** (−1.28, −1.43, −1.76, −1.78 eV for **2a**–**5a**, respectively, versus −0.37 eV for **1a**). Altogether, these data suggest that compounds **2a**–**5a** are stronger π-acceptors but weaker σ-donors than CAAC **1a**. In this series, carbene **1a** also has the widest HOMO–LUMO gap of 5.39 eV, indicating the CAACs’ general stability and their widespread use as σ-donating ligands.^1^ For the other compounds **2a**–**5a**, HOMO–LUMO gap values of 4.84 (**2a**), 4.73 (**3a**), 4.25 (**4a**), and 4.10 eV (**5a**) were obtained. As such, upon descending group 14, the HOMO–LUMO gap becomes narrower due to the increase of the HOMO energies and decrease of the LUMO energies. The increasing destabilization of the HOMO for the heaviest species in the group not only indicates increasing reactivity for the molecules but also decreasing stability.

**Figure 3:**
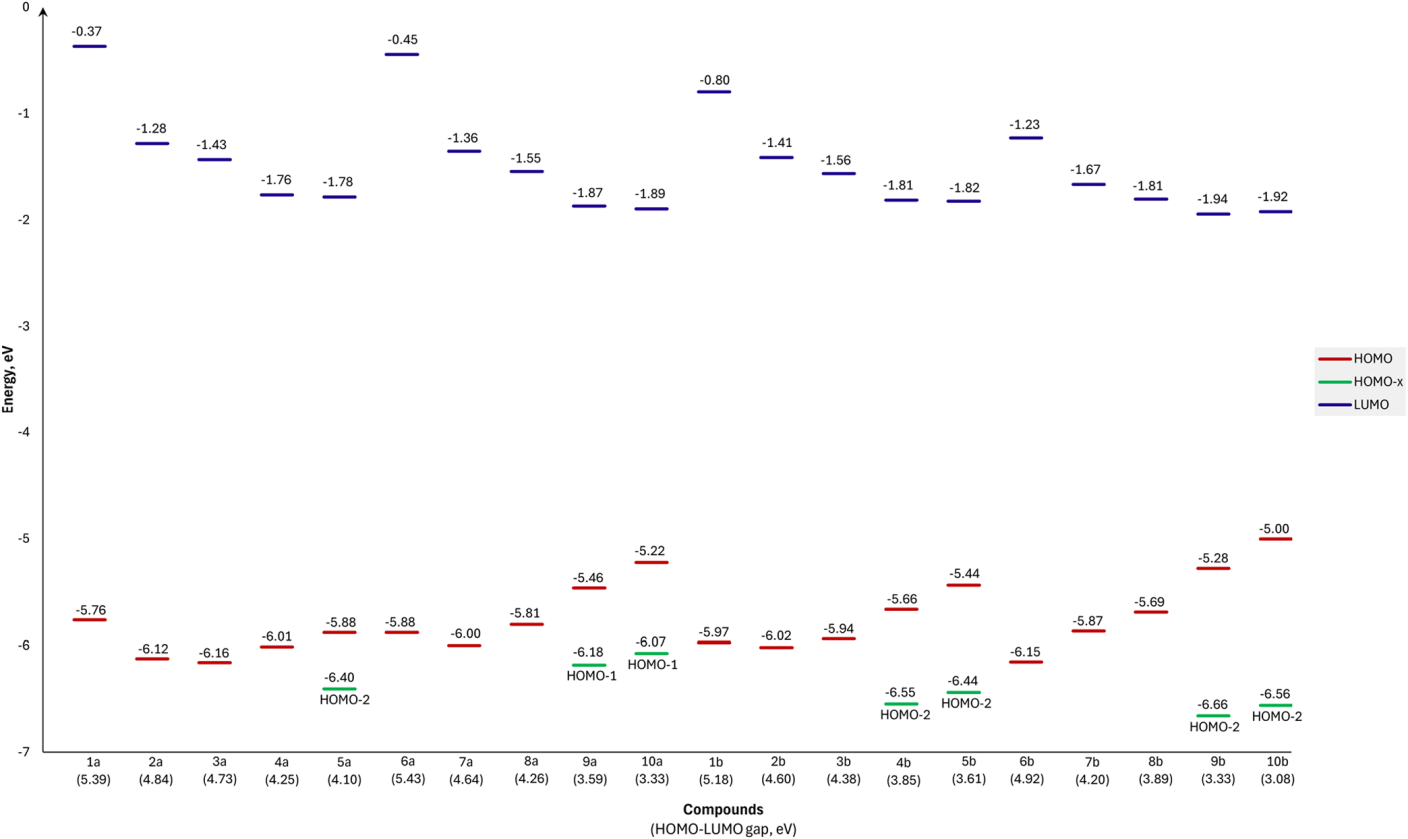
Calculated HOMO, HOMO–x, and LUMO energy levels for species **1–10** (HOMO-LUMO gaps (eV) in brackets).

Similar FMO characteristics were obtained for the remaining investigated compounds **6a**–**10a** and **1b**–**10b** (see SI). This is as expected due to the observed incremental changes in the bonding parameters due to ligand sterics and/or electronics discussed above. All 8 investigated CAASn and CAAPb derivatives exhibit stable lone pair orbitals (HOMO–1 or HOMO–2), consistent with the so-called inert pair effect.^32^ The HOMO for unsaturated derivatives **9** and **10** is composed of the π-bond in the heterocycle backbone, which might indicate competitive reactivity pathways for the unsaturated CAAE derivatives. In fact, unsaturated NHCs have been shown to react through the double-bonded 4/5-carbon position rather than the carbenic carbon in the 2-position.^33,34^ Finally, the HOMO–LUMO gaps are the narrowest for the CAASn and CAAPb analogs, and the smallest gap of 3.08 eV was observed for CAAPb **10b**.

The chemistry of singlet silylene and germylene compounds is steadily increasing in terms of structure and reactivity.^5,35–37^ To date, none of the saturated derivatives discussed in this study have been reported; however, a compound similar to **2a**, CAASi **I**, with an adamantyl substituent attached to the nitrogen atom, has been isolated (Figure 1b).^6^ Compound **I** exhibited thermal stability while maintaining its reactivity. In addition, the inspection of the frontier molecular orbitals, calculated at the B3PW91-D3/6-31G­(d) level of theory, revealed that the HOMO (−5.56 eV) and LUMO (−1.16 eV) correspond to the Si-centered lone pair orbital and empty 3p orbital, respectively, similar to those calculated here for **2a**. The bulky adamantyl group on the nitrogen atom donates more electron density to the Si center than the Dipp group in **2a**, thus increasing the HOMO and lowering the LUMO energy of **I** relative to **2a**. This results in an increase of the reactivity of **I** compared to that of **2a** (see below). The computed electronic structure of **8a** is in excellent agreement with the data obtained for isolated CAAGe **II**.^7^ Here, the FMO energies are well reproduced despite the use of different levels of theory (B3LYP/6–31G­(d,p) in the case of **II**). Germylene **II** was also shown to be reactive toward small molecules such as N_2_O. The third example, stannylene **III**, has a three-coordinate Sn-center and a ligand framework very different from that of the CAASns studied here.^13^ In addition, no further reactivity was reported for compound **III**. In general, our computations are in good agreement with the results obtained previously for analogous CAASi and CAAGe compounds.^14,15^

### Reactivity toward Dihydrogen

Group 14 tetrylenes have been shown to react with important small molecules, such as H_2_, NH_3_, and CO_2_.^5,36,38^ For example, the reaction between a tetrylene and molecular hydrogen can be described formally as an oxidative addition of the small molecule, and the product of type R_2_E­(H)­H with a tetrahedral E­(IV) center representing the thermodynamic sink structure. Small-molecule activation reactions are generally stoichiometric, but a clear impetus exists to create a system in which the reaction is close to thermoneutral to develop transition metal-free catalytic applications. The above electronic structure analysis revealed that the investigated compounds **1–10** could be synthetically feasible, at least for E = Si and Ge. The Pb- and Sn-derivatives might require additional electronic stabilization for experimental realization, but then lack the appropriate FMO compositions for further reactivity. Nevertheless, we were interested in studying the CAAEs’ reactivity toward H_2_, CO_2_, and NH_3_ and comparing the reaction pathways and energetics to those of the parent CAACs.

First, the reactivities of **1–10** toward H_2_ were investigated. The expected oxidative addition products and transition state structures were optimized to examine the Gibbs free energies of activation and overall energetics of the reactions. Overall, the mechanism for dihydrogen activation by the CAAE compounds was found to resemble that established for the parent carbene CAAC, which proceeds via a concerted transition state.^4,39,40^ Dihydride product formation occurs by heterolytic cleavage of the H–H bond, facilitated by the donation of electron density from the group 14 element lone pair and simultaneous acceptance of electron density by the empty p-orbital, as expected.

Generally, the narrowing of the HOMO–LUMO gap as one descends group 14 could indicate an increase in the reactivity of the lone pair at the expense of stability (Figure 3). As the lone electron pair orbital (HOMO) and empty p-orbital (LUMO) become closer in energy, the tendency to form the oxidative addition product upon reaction with H_2_ should become more favorable. This is not expected to apply to CAASn and CAAPb derivatives due to the increased stability and nonbonding nature of the lone electron pair. As such, the calculated reaction profiles for **1a**–**5a** (Figure 4) corroborate the expected reactivity. The reaction free energies and activation barriers indicate the decreasing feasibility of dihydrogen activation as one moves down the group. The reaction free energy was calculated to be exergonic for **1a**–**3a** (Δ*G*_r_ = −38.2 (**1a**), −27.2 (**2a**), and −8.7 kcal mol^–1^ (**3a**)) and endergonic for **4a** and **5a** (Δ*G*_r_ = 7.5 and 32.4 kcal mol^–1^, respectively). CAAEs **2a**–**5a** exhibit significantly higher activation barriers compared to **1a**, which is attributed to the heavier analogs’ reduced Lewis basicity, as corroborated by the lower energies of the σ-symmetric lone pair orbitals. In addition, the transition states are destabilized for heavier congeners due to the increased size of the element E and spatial disparity of the valence orbitals (no possibility of hybrid orbitals for E = Ge–Pb). As such, the activation energies for derivatives **2a**–**5a** were found to be high (Δ*G* ^‡^ = 40.8 to 72.5 kcal mol^–1^), and thus the reactions are unlikely to occur, at least at room temperature. Especially in the case of Sn- and Pb-derivatives **4a** and **5a**, respectively, the unfavorable reactivity may be attributed to the nonbonding nature and stability of the ns orbitals, as the promotion energy is not fully compensated by the release of energy from the formation of new bonds.^31^ As a result, the oxidative addition product with element E in the oxidation state +4 is much more difficult to obtain as the element E becomes heavier.

**Figure 4:**
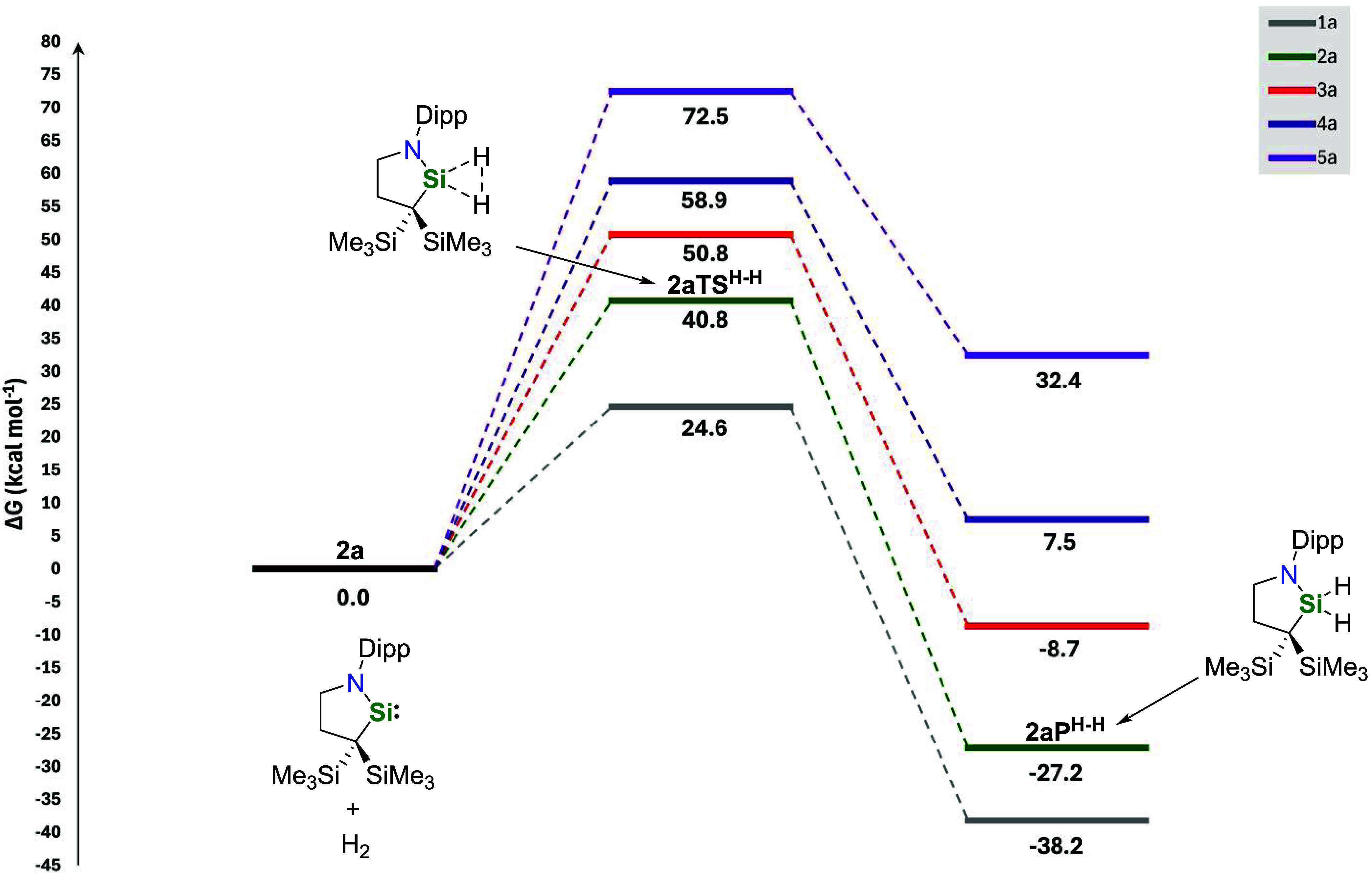
Calculated energy profile diagram for the activation of H_2_ by **1a**–**5a**.

Further analysis of the interactions between the CAAE derivatives and H_2_ was conducted using energy decomposition analysis coupled with natural orbitals for the chemical valence (EDA-NOCV) approach.^41^ The transition state geometry of H_2_ activation by **1a**–**5a** (i.e., structures **1–5aTS** ^**H–H**^ in Figure 4) was divided into two neutral fragments, namely CAAE and H_2_, and the bonding characteristics between these fragments were studied. The breakdown of the numerical results of the analysis (see SI, Table S2) reveals that the orbital interaction term constitutes more than half of the attractive forces for all E = C–Pb, being the greatest for CAAC **1a** (65.7%) and decreasing to 53.4% in the case of the heaviest CAAPb **5a**, corroborating the above statement of decreasing favorable orbital interactions between the reactants upon descending the group. Moreover, more than 90% of all orbital interactions can be described by two pairwise orbital interactions, Δ*E*_Orb(1)_ and Δ*E*_Orb(2)_, with ca. 75% contribution from Δ*E*_Orb(1)_ for **1a** and **3a**–**5a**, and 59% for **2a**. Visual inspection of the deformation densities confirms the nature of the most prominent orbital interactions to be the charge flow from the CAAE lone pair orbital to the σ*-orbital of H_2_ (Δ*E*_Orb(1)_) with secondary interactions from the HOMO of H_2_ to the empty p-orbital LUMO of CAAE (Δ*E*_Orb(2)_).

The reaction free energies for **6a**–**9a**, as well as the phenyl-substituted analogs **1b**–**10b**, are comparable to those of the saturated analogs **1a**–**5a** and are thus not discussed in detail here (see SI). The relatively small differences between the FMOs and comparable activation energies in the saturated and unsaturated series of analogs suggest that the activation of H_2_ is negligibly affected by the saturation of the backbone or by the substituent R. In contrast, the process for the oxidative addition of dihydrogen to CAAPb **10a** was found to be unfeasible. No transition state analogous to **5a** could be located for unsaturated **10a**. Instead, a pathway in which the Pb–C bond is cleaved to yield an acyclic species was identified. However, this process involves a prohibitively high activation energy (Δ*G* ^‡^ = 52.3 kcal mol^–1^) (see SI). Based on these calculations, the activation of molecular hydrogen by CAAPb is not probable, and the likelihood of other competing mechanisms, such as decomposition, could proceed with lower barriers and intermediate structures. This result highlights the reluctance of the Pb 6s electrons to participate in the bonding.

### Reactivity toward Ammonia

CAACs are known to undergo oxidative addition with ammonia to produce the amido-hydride of type CAAC­(H)­NH_2_.^42^ Similarly, a few tetrylenes^38,43,44^ have been reported to showcase similar reactivity; however, ammonia activation is still quite rare due to the strength of the N–H bond (ca. 100 kcal mol^–1^).^45^ Transition metal complexes, for example, prefer the formation of a simple Werner coordination complex over oxidative addition.^46–49^ In our previous study, we investigated the possibility of ammonia activation by group 13 and 14 compounds, and concluded that for those β-diketiminate-supported p-block compounds, the preferred reaction mode is indeed oxidative addition.^49^ Hence, we were interested in studying whether the same is true for the CAAE derivatives. Since no significant differences between the saturated (**1–5**) and unsaturated (**6–10**) or TMS (**a**) vs Ph (**b**)-substituted CAAEs could be identified, only saturated analogs **1a**–**5a** are discussed herein.

Similar to the dihydrogen activation study above, the products and transition state geometries were optimized. Ammonia activation was first considered to proceed analogously to H_2_ activation via the heterolytic cleavage of one of the N–H bonds. However, for all heavier tetrylenes, it was found that NH_3_ reacted with CAAE by first forming an ammine adduct (Figure 5). Only then can N–H bond cleavage occur to yield the amido-hydride product CAAE­(H)­NH_2_. The Werner-type adduct is a stable intermediate for all the calculated species, as local minimum structures are located on the potential energy surface. The optimized E–N bonds in the adducts are, in some cases, long (Table S10), but shorter than the calculated sum of the respective van der Waals radii,^50^ suggesting that interactions are present between the E center and N atom of ammonia. Interestingly, a transition state for adduct formation was only observed for **1a** with a barrier of 42.3 kcal mol^–1^ (Figure 5), and the adduct **1aInt** ^**N–H**^ was calculated to be 40 kcal mol^–1^ higher in energy than the free starting materials. These high activation and adduct energies are consistent with the strong nucleophilic character of CAACs, as described by Bertrand.^42^ The transition state for N–H bond cleavage by **1a** was calculated to be 32.2 kcal mol^–1^, and the intrinsic reaction coordinate (IRC) calculations confirmed that the oxidative addition of ammonia occurred directly from the free carbene and did not involve the adduct intermediate. Similar to the H_2_ mechanism, the N–H bond breaking becomes more energy demanding as one descends group 14 (Δ*G* ^‡^(**1a**) = 32.2 kcal mol^–1^ to Δ*G* ^‡^(**5a**) = 74.4 kcal mol^–1^). In addition, the reaction is endergonic for CAASn and CAAPb. Very similar energy profiles were obtained for the Ph-substituted series **1b**–**5b**, again confirming the negligible effect of the identity of substituent R (see SI).

**Figure 5:**
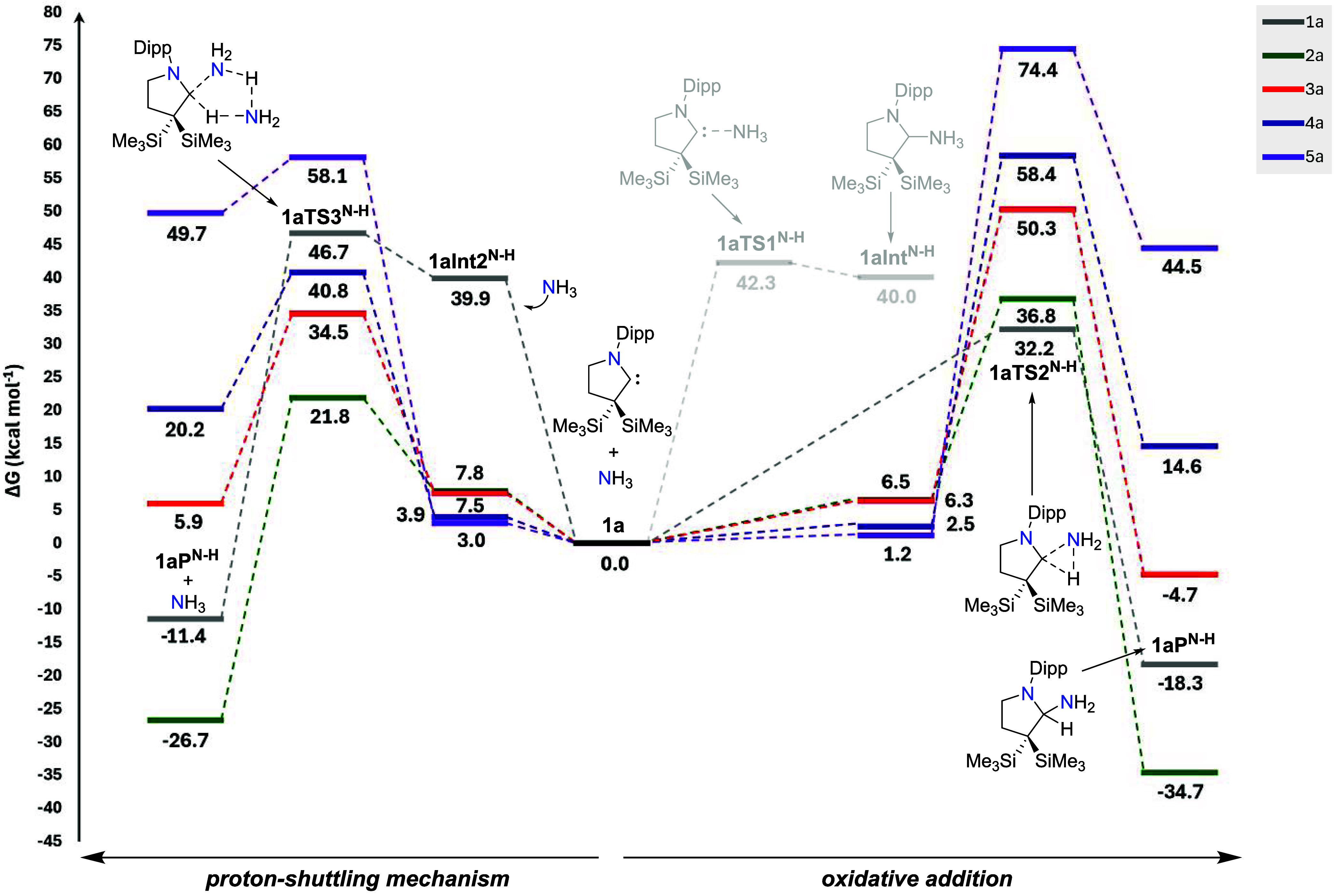
Energy profile diagram for the activation of NH_3_ by **1a**–**5a** via oxidative addition or proton-shuttling mechanism.

Several other studies have identified a potential proton-shuttling mechanism for ammonia activation that may be more likely to occur.^44,51–53^ In this mechanism, two ammonia molecules participate in the reaction by first forming an adduct with one ammonia molecule and CAAE (Scheme 1). The participation of a second molecule of ammonia is required to transfer one of its hydrogens to the E atom center. Simultaneously, the second NH_3_ moiety acquires a proton from the coordinated ammonia to regenerate the molecule and yield an amido-hydride product. Indeed, this sequence lowers the barriers for CAAEs **2a**–**5a** compared to the single ammonia mechanism (Figure 5). For example, in the case of the CAASi **2a**, the associated barrier is 15.0 kcal mol^–1^ lower when considering the proton-shuttling mechanism. For CAAC **1a**, the preferred mode of ammonia activation is direct oxidative addition, probably due to the better matching of the FMOs and hybridization of the s- and p-orbitals, as discussed above.^42^

**Scheme 1:**
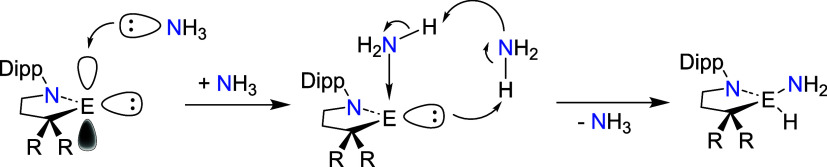
Proton-Shuttling Mechanism Involving Two NH_3_ Molecules for the Oxidative Addition of the N–H Bond in NH_3_ at CAAE Group 14 Species

As an example, for CAAC **1a**, the optimized transition state structure of the proton-shuttling mechanism (**1aTS3** ^**N–H**^) features a five-membered heterocyclic structure (Figure S23). In this structure, the N–H bond of the bound ammonia is polarized (|*q*_M_| = 0.42, *q*_M_(N) = −0.19, and *q*_M_(H) = +0.23) and lengthened (1.228 Å). Similarly, the N–H bond in the second ammonia molecule is lengthened (1.200 Å) as H approaches the E-bound nitrogen. Altogether, two N–H bonds are cleaved, and two new (one N–H and one E–H) bonds are formed in this concerted transition state. In contrast, the heavier analogs, such as **4a** and **5a**, exhibited a late transition state, likely due to the larger size of Sn and Pb and the nonbonding nature of the 5s and 6s electrons compared to the lighter congeners. The proton of the second ammonia molecule must travel farther in the Sn- and Pb-analogs, as is evident from the wide N–E–H angles in the TS structures (Table S11). The optimized TS structure for **4a** indicates that the first sequence of N–H bond breaking and a new N–H bond forming has already occurred, as the Sn-bound N–H bond length is 2.166 Å and the new H–N bond length is 1.034 Å. Thus, the proton-shuttling mechanism for CAASn and CAAPb could be described as a more stepwise process than concerted. Although the first step in the proton-shuttling mechanism is the proton transfer from bound ammonia to free ammonia to form NH_4_^+^, no local minimum for this intermediate was found. It is likely that this first step occurs without a significant barrier, as it immediately relaxes to form a stable ammonia adduct. Thus, the only observed transition state is the second step in the process, in which the second ammonia molecule transfers a proton to the E center, forming a new E–H bond.

### Reactivity toward Carbon Dioxide

A number of silylenes and germylenes have been reported to react with CO_2_.^5,40,54^ In particular, the activation of CO_2_ by silylenes produces ylidic Si=O bonds that can further form silanones, dinuclear Si­(IV) compounds, and other higher coordination Si­(IV) species.^40,55–58^ In this regard, we studied the potential of CAAEs **1–10** for the activation of carbon dioxide. Similar to the H_2_ and NH_3_ reactions, the CO_2_ activation products were first targeted. Perhaps unsurprisingly, the activation of CO_2_ is not feasible with any of the Ph-substituted derivatives **1b**–**10b** or with the Ge, Sn, or Pb derivatives **3a**–**5a** and **8a**–**10a**, as no stable compounds with E-CO_2_ interactions were found, although several possible CO_2_ activation products can be envisaged due to the electron-donating and -accepting properties of CO_2_.^59^ The lightest CAAC and CAASi compounds, however, were observed to interact with CO_2_. Here, the mechanism of CO_2_ activation by carbenes **1a** and **6a** involves the transfer of electron density from the lone pair of the E atom to the antibonding 2π_u_* orbital of CO_2_, forming a CO_2_ adduct via the CO_2_ carbon, as reported previously (Figure 6a).^5,60^ The Si-analogs **2a** and **7a** coordinate CO_2_ via both carbon and oxygen, forming η^2^-CO coordination (Figure 6b).^59,61,62^ This coordination mode is mainly observed for transition metals, with the most prevalent being Aresta’s complex Ni­(CO_2_)­(PCy_3_)_2_ (Cy = cyclohexyl).^63^ This mode involves the interaction of the 1π_g_ orbital of CO_2_ with the vacant p-orbital of atom E, while E simultaneously transfers electron density from its lone pair orbital to the vacant antibonding orbital of CO_2_, forming a three-membered ring structure. These orbital interactions weaken the multiple bonds in linear CO_2_, causing the bending of the molecule. In support of this view, EDA-NOCV calculations were performed on the transition state geometries of CO_2_ activation by **1a** and **2a**. The results indicate that adduct formation between CAAC **1a** and CO_2_ is governed by electrostatic rather than orbital interactions (see SI). In contrast, the orbital interactions outweigh the electrostatic interactions for **2a**. A closer look at the deformation densities confirms that the most important orbital contribution originates from the above-mentioned CAAE HOMO to CO_2_ LUMO interactions for both **1a** and **2a**.

**Figure 6:**
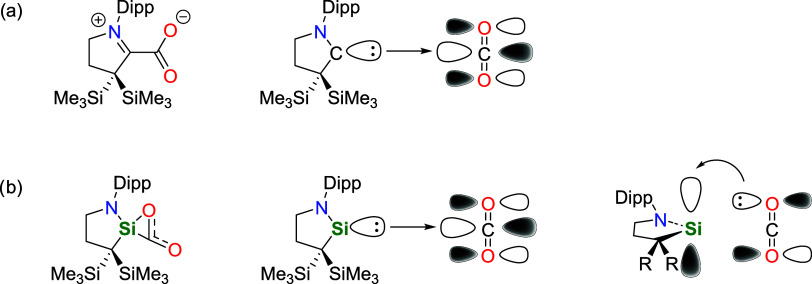
(a) η^1^-C CO_2_ coordination mode exhibited by **1a** and **6a** (left) and the relevant FMO interactions (right). (b) η^2^-CO CO_2_ coordination mode exhibited by **2a** and **7a** (left) and the relevant FMO interactions (right). Only saturated analogs **1a** and **6a** are shown.

The Gibbs free energies of CO_2_ activation are exergonic for **1a** (Δ*G*_r_ = −13.4 kcal mol^–1^), **2a** (Δ*G*_r_ = −3.9 kcal mol^–1^), and **6a** (Δ*G*_r_ = −14.1 kcal mol^–1^), and thermoneutral for **7a** (Δ*G*_r_ = 0.9 kcal mol^–1^) (Figures 7 and S33). The corresponding activation energies were found to be small for the CAAC analogs **1a** (Δ*G* ^‡^ = 5.4 kcal mol^–1^) and **6a** (Δ*G* ^‡^ = 4.1 kcal mol^–1^) and modest for CAASi analogs **2a** (Δ*G* ^‡^ = 16.1 kcal mol^–1^) and **7a** (Δ*G* ^‡^ = 19.3 kcal mol^–1^), suggesting the feasibility of the synthesis. When moving from C to Si (**1a** to **2a**), the activation energy increases from 5.4 to 16.1 kcal mol^–1^. This trend is also observed between **6a** and **7a**, which is corroborated by the electronic structures of the C-analog **1a** and the Si-analog **2a**. CAACs contain a stabilized HOMO and a destabilized LUMO, thus a large HOMO–LUMO gap, which makes the compound a good σ-donor that can form an adduct with CO_2_. On the other hand, the CAASi compounds benefit from a lower-lying LUMO with respect to carbenes, which facilitates electron donation from the CO_2_ lone pair orbital to the empty p-orbital of silicon.

**Figure 7:**
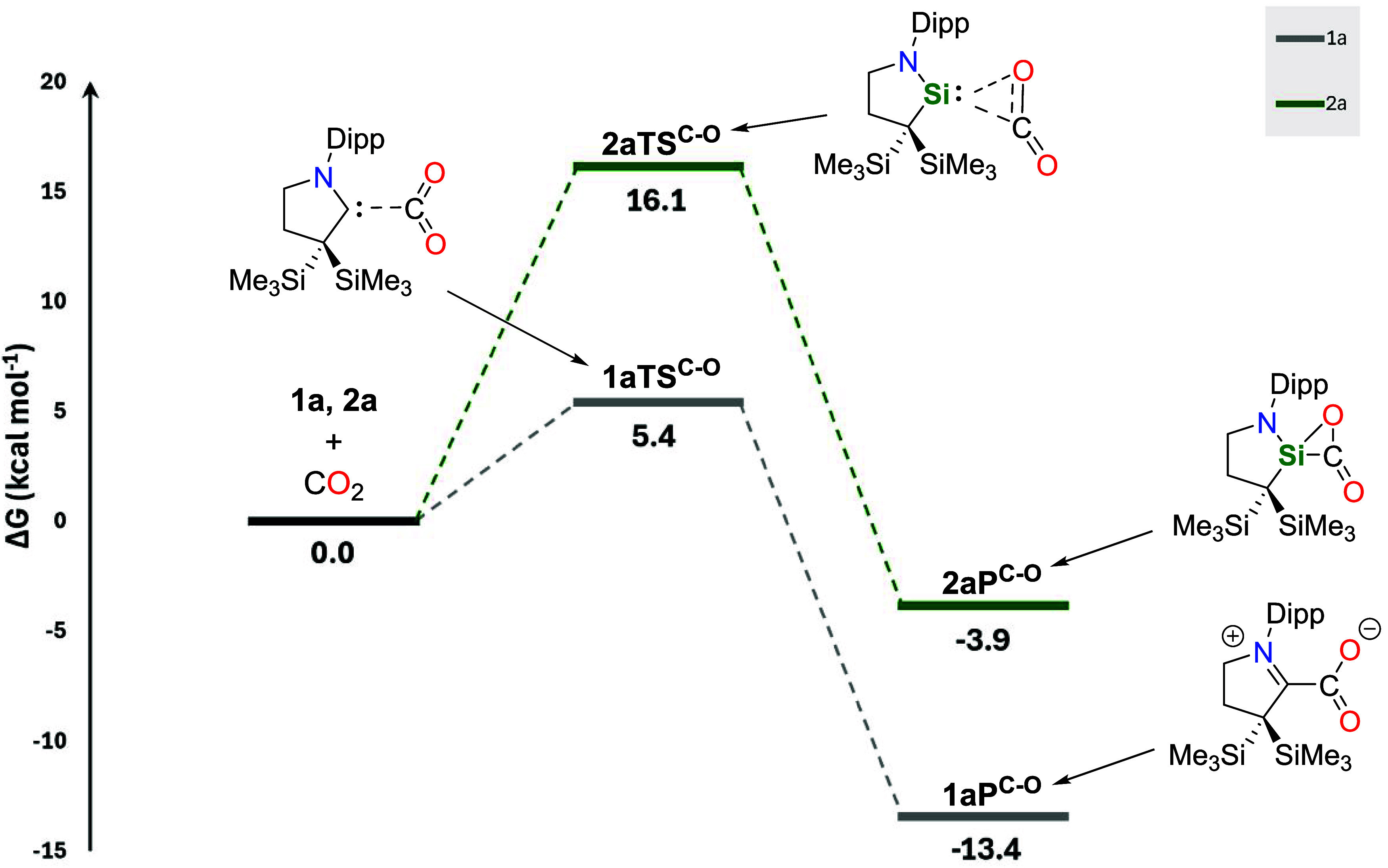
Calculated energy profile diagram for the activation of CO_2_ by **1a** and **2a**.

The different bonding modes of CO_2_ are likely determined by the basicity and electrophilicity of atom E.^62^ Both C and Si centers in CAAEs have a coordination vacancy that can bind CO_2_ through carbon directly or through both carbon and oxygen. Since the energy of the lone electron pair orbital (HOMO) of **1a** is greater than that of **2a** (−5.76 versus −6.12 eV), the CAAC compound is more nucleophilic and will react with CO_2_ more readily than CAASi. Moreover, as **2a** is less nucleophilic but more electrophilic than **1a**, CAASi is more likely to form an η^2^-CO coordination complex. The unsaturated series of these analogs (**6a** and **7a**) also demonstrated the same trends (see SI). Carbenes **1a** and **6a** both have low activation energies, 5.4 and 4.1 kcal mol^–1^, respectively, which demonstrates that the modification of the backbone saturation had little impact on the activation of CO_2_. Additionally, the low energy barrier suggests that the reaction can proceed reversibly at room temperature, in which rapid activation and dissociation of CO_2_ can occur, known as thermal lability.^62^ The reversibility and favorable reaction thermodynamics showcased by the Si-analogs, in addition to the carbenes, should be investigated in depth experimentally, as this could lead to new potential applications in CO_2_ catalysis and valorization.

## Conclusion

A study employing density functional theory (DFT) was performed to gauge the feasibility of heavier analogs of cyclic (alkyl)­(amino)­carbenes' (CAACs) reactivity toward dihydrogen, ammonia, and carbon dioxide. A library of CAAE compounds **1–10** was optimized to shed light on their geometries and electronic structures, especially FMO energies and compositions, and trends in molecular stability and reactivity. The substitution of trimethylsilyl versus phenyl groups in the quaternary carbon position revealed that the substituent played only a small role in the stability and reactivity of the species; both derivatives had comparable characteristics toward small-molecule activation. Similarly, saturation of the backbone resulted in negligible differences in the thermodynamic reaction parameters between the analogs. Next, the CAAEs’ abilities for the activation of H–H, N–H, and C–O bonds were investigated. Their reactivity toward dihydrogen was as expected, following a nucleophilic activation and hydride transfer mechanism, leading to the oxidative addition product. The thermodynamic parameters of these reactions indicated that dihydrogen activation is likely not feasible at ambient conditions for any analog, and the activation energies were observed to increase as one descends the group. Similarly, the reactivity toward ammonia was investigated, and, as expected, the oxidative addition of ammonia was shown to be unfavorable and less feasible as the element E gets heavier. Ammonia activation was also investigated via a proton-shuttling mechanism involving two equivalents of NH_3_, which revealed that the mechanism was more favorable than direct oxidative addition with decreased activation barrier heights. Finally, the activation of CO_2_ was investigated and was found to resemble that of transition metal complexes for CAACs and CAASis. The computed low barrier heights indicated potentially thermally labile compounds that could rapidly activate and dissociate CO_2_. Overall, small-molecule activations employing heavier CAAEs were calculated to have higher activation energies than the lightest CAAC analogs, indicating the requirement of elevated reaction temperatures or otherwise more forcing conditions. The calculated energy barriers and reaction free energies suggest that the activation of ammonia via the proton-shuttling mechanism may be plausible for C, Si, and Ge, as well as the activation of CO_2_ for C and Si, warranting further experimental studies to be conducted.

## Supplementary Material





## Notes

Optimized coordinates of the compounds (ZIP)
Comparison of experimental and calculated bond parameters (Table S1); Frontier molecular orbitals of CAAE compounds **1**–**10** (Figures S1–S20); EDA-NOCV analysis (Tables S2, S3; Figures S21, S22); summary of reaction thermodynamics (Tables S4–S7); transition state structures (Figure S23; Tables S8–S13); calculated energy diagrams (Figures S24–S33) (PDF)

## Abbreviations

FMO: frontier molecular orbital
HOMO: highest occupied molecular orbital
LUMO: lowest unoccupied molecular orbital
Ph: phenyl
TMS: trimethylsilyl, SiMe_3_
