# Supplementary material for: Activation of Small Molecules by Heavier Analogs of Cyclic(Alkyl)(Amino)Carbenes (CAACs): A DFT Study
Source: Inorg Chem. 2026 Jul 10;65(29):16713–23. doi: 10.1021/acs.inorgchem.6c01118 (PMC13418173; doi:10.1021/acs.inorgchem.6c01118)
Supplement: Supplementary file 2 [file ic6c01118_si_002.pdf]

Supporting Information

for

Activation of small molecules by heavier analogs of cyclic (alkyl)(amino) carbenes (CAACs): a DFT study

by

Britney Cai[a,b] and Petra Vasko*[a]

[a]*Department of Chemistry, P. O. Box 55 (A. I. Virtasen Aukio 1), 00014 University of Helsinki, Finland. E-mail: petra.vasko@helsinki.fi*

[b]*Department of Chemistry, 2500 University Dr, Calgary, NW T2N 1N4 University of Calgary, Alberta, Canada*

Table of Contents

1. Comparison of experimental and calculated bond parameters 3
2. Frontier molecular orbitals of 1-10 3
3. EDA-NOCV analysis 10
4. Summary of reaction thermodynamics 14
5. Transition state structures 15
6. Calculated energy diagrams 18
7. References 23

1. Comparison of experimental and calculated bond parameters

Table S1. A comparison of experimental and computational bond parameters and HOMO/LUMO energies.

Compound	E-N (Å)	E-C (Å)	N-E-C (°)	HOMO (eV)	LUMO (eV)	HOMO-LUMO gap (eV)	Level of Theory	Ref.
**^{Me}cAAC (exp.)**	1.3053(13)	1.5262(13)	105.92(8)	-	-	-	-	[1]
**^{Me}cAAC (calc.)**	1.301	1.513	106.7	-5.81	-0.36	5.45	(PCM=THF)PBE0-D3(BJ)/Def2-TZVP	[2]
**1a (calc.)**	1.320	1.486	106.5	-5.76	-0.37	5.39	PBE0- D3(BJ)/Def2-SVP	This work
**I (exp.)**	1.7143(15)	1.914(2)	92.64(8)	-	-	-	-	[3]
**I (calc.)**	1.740	1.925	91.5	-5.56	-1.16	4.40	B3PW91-D3/6-31G(d)	[3]
**2a (calc.)**	1.739	1.928	90.4	-6.12	-1.28	4.84	PBE0- D3(BJ)/Def2-SVP	This work
**II (exp.)**	1.859(11)	1.998(14)	86.5(5)	-	-	-	-	[4]
**II (calc.)**	1.880	1.987	86.8	-5.39	-1.44	3.95	B3LYP/6-31G(d,p)	[4]
**8a (calc.)**	1.879	2.012	85.5	-5.81	-1.55	4.26	PBE0- D3(BJ)/Def2-SVP	This work
**CAASi (calc.)**[a]	1.742	1.900	91.5	-6.13	-1.50	4.63	(PCM=toluene)M06-D3/Def2-TZVP	[5]
**CAAGe (calc.)**[a]	1.869	2.012	87.7	-6.28	-1.88	4.40	(PCM=toluene)M06-D3/Def2-TZVP	[5]
**3a (calc.)**	1.839	2.028	87.4	-6.16	-1.43	4.73	PBE0- D3(BJ)/Def2-SVP	This work

[a]N-bound substituent Ph

2. Frontier molecular orbitals of 1-10

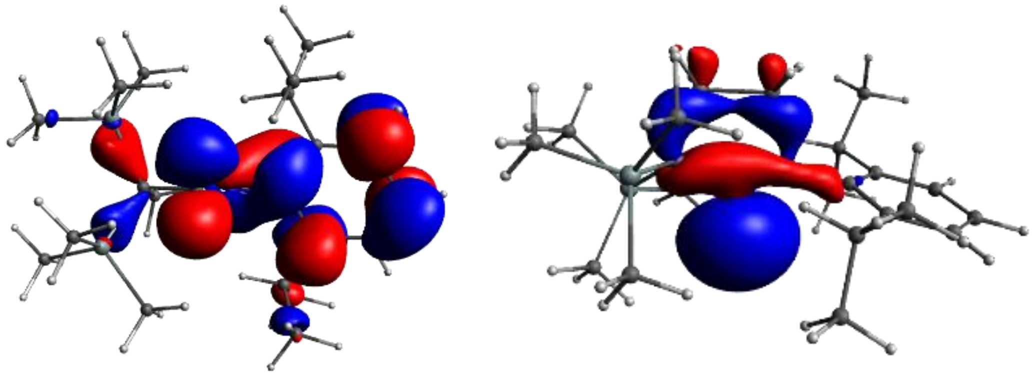

Figure S1. The LUMO (left) and HOMO (right) of **1a**. Isovalue set at 0.04 a.u.

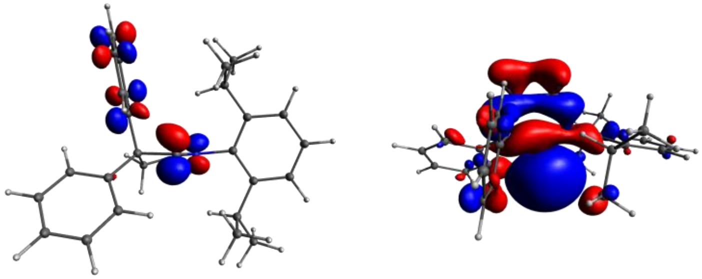

Figure S2. The LUMO (left) and HOMO (right) of **1b**. Isovalue set at 0.075 a.u.

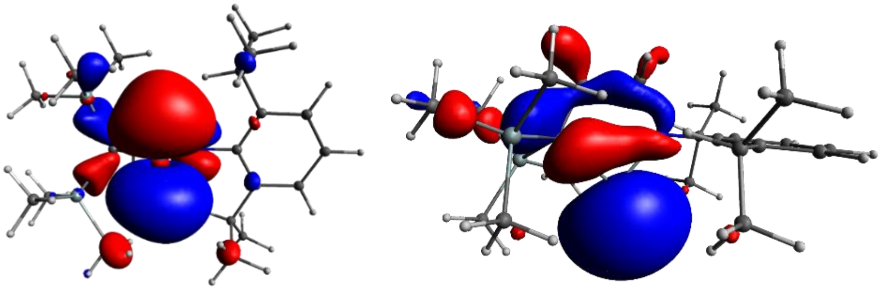

Figure S3. The LUMO (left) and HOMO (right) of **2a**. Isovalue set at 0.03 a.u.

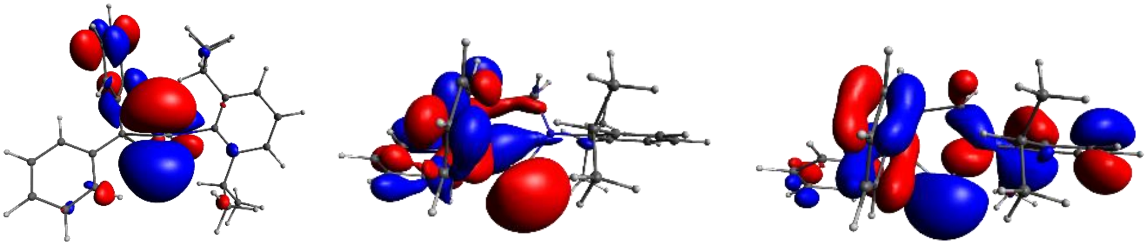

Figure S4. The LUMO, HOMO, and HOMO-1 (from left to right) of **2b**. Isovalue set at 0.04 a.u.

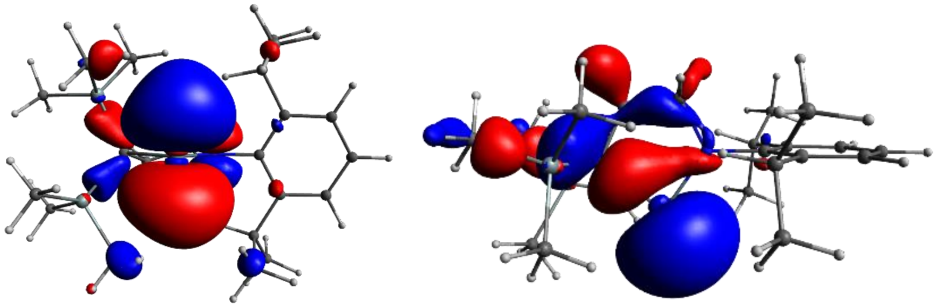

Figure S5. The LUMO (left) and HOMO (right) of **3a**. Isovalue set at 0.03 a.u.

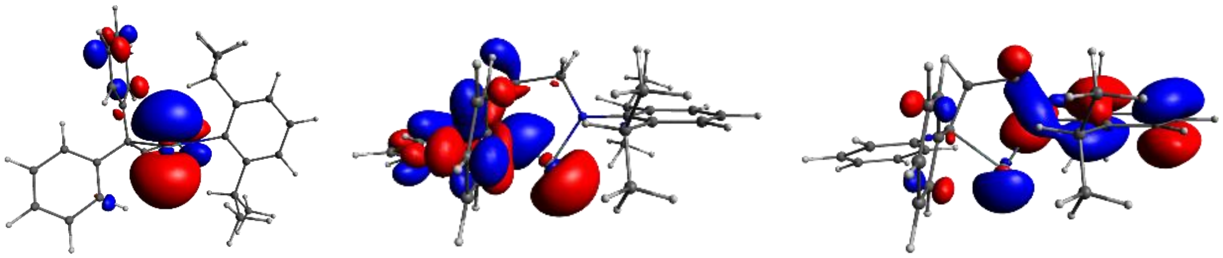

Figure S6. The LUMO, HOMO, HOMO-1 (from left to right) of **3b**. Isovalue set at 0.04 a.u.

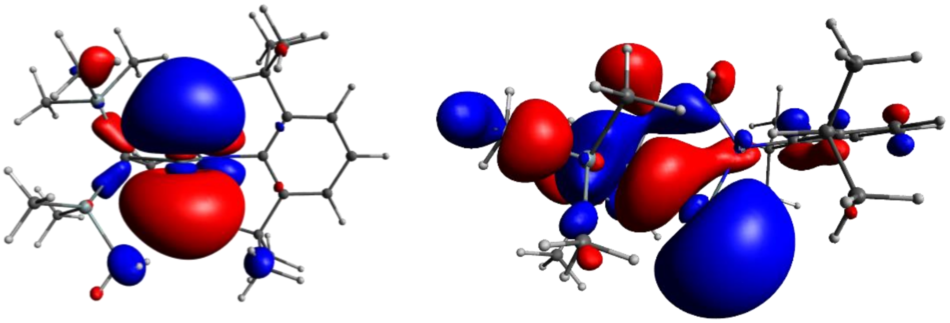

Figure S7. The LUMO (left) and HOMO (right) of **4a**. Isovalue set at 0.02 a.u.

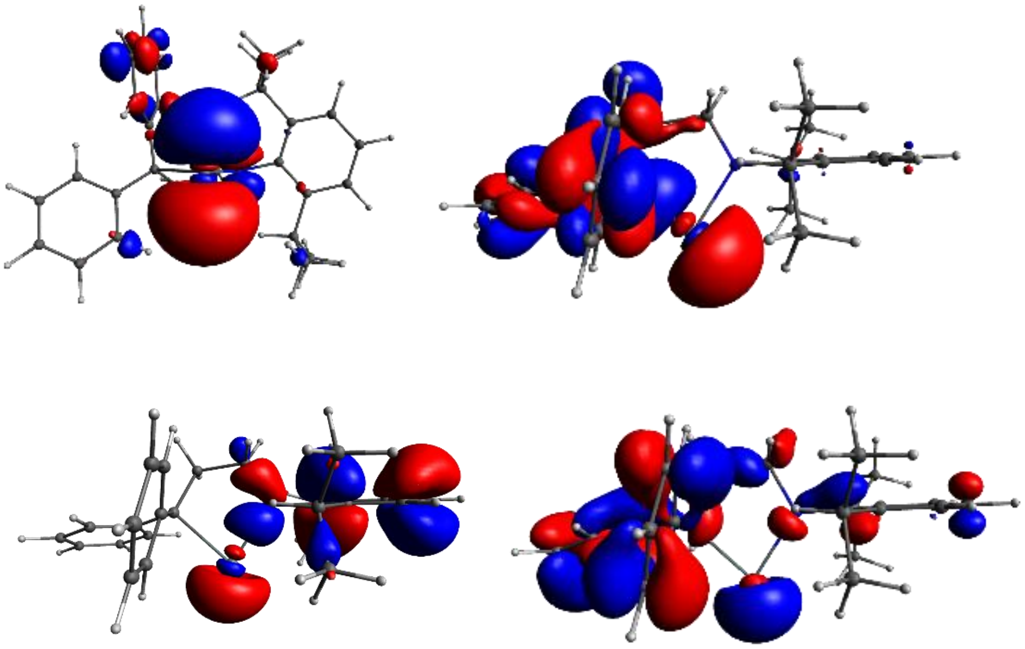

Figure S8. The LUMO (upper left), HOMO (upper right), HOMO-2 (lower left) and HOMO-4 (lower right) of **4b**. Isovalue set at 0.03 a.u.

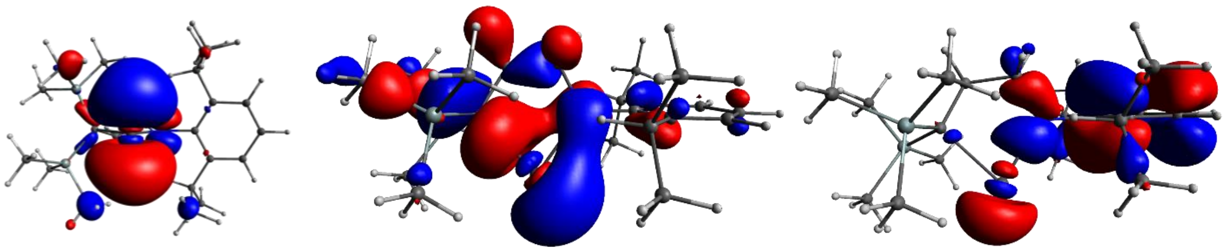

Figure S9. The LUMO (left), HOMO (middle) and HOMO-2 (right) of **5a**. Isovalue set at 0.03 a.u.

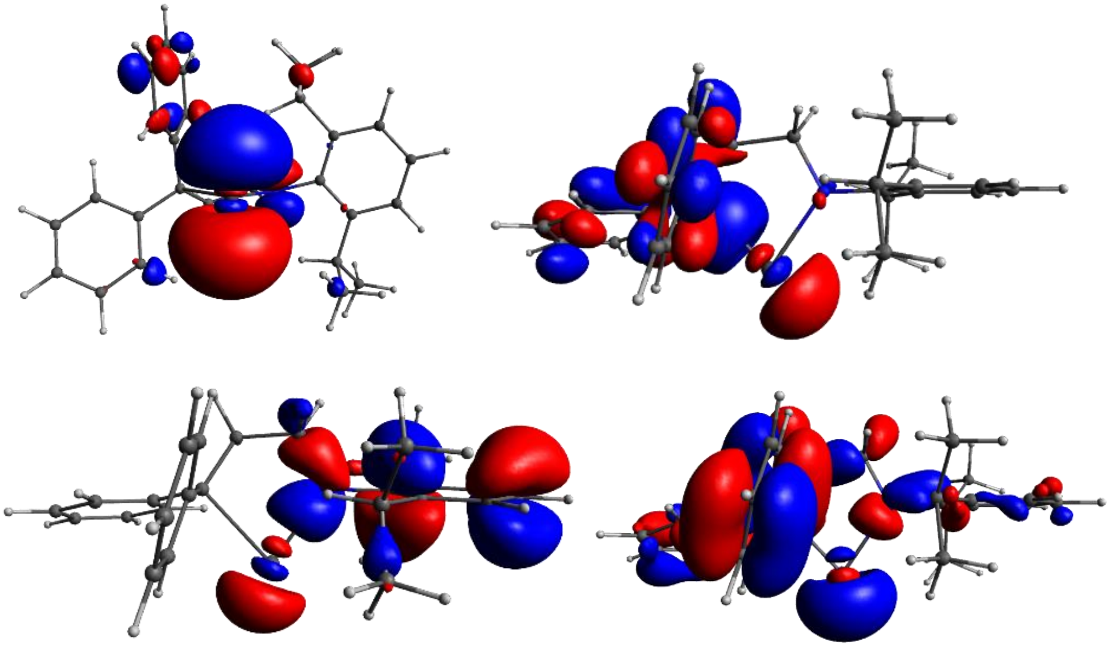

Figure S10. The LUMO (upper left), HOMO (upper left), HOMO-2 (lower left) and HOMO-7 (lower right) of **5b**. Isovalue set at 0.03 a.u.

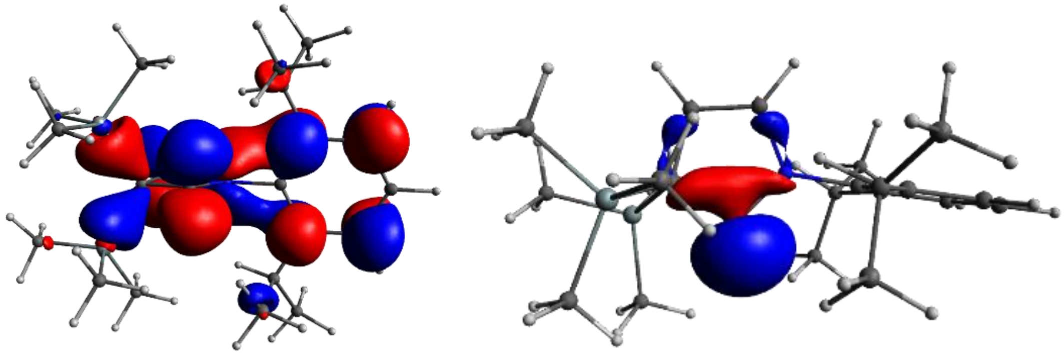

Figure S11. The LUMO (left) and HOMO (right) of **6a**. Isovalue set at 0.03 a.u.

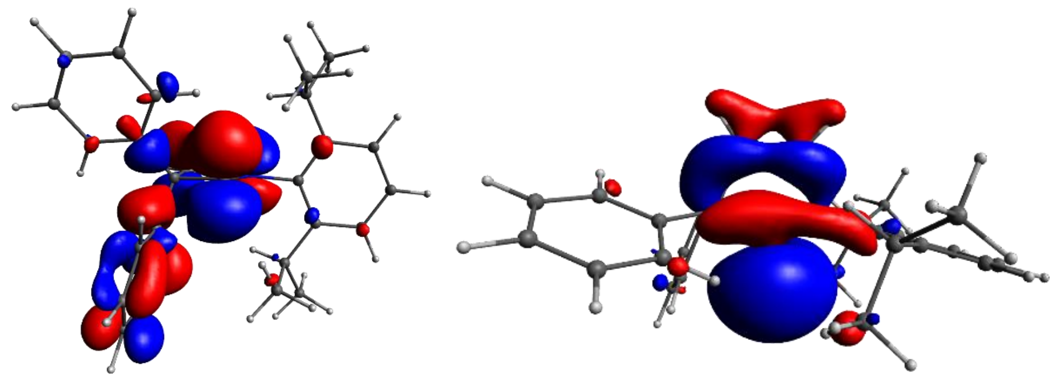

Figure S12. The LUMO (right) and HOMO (left) of **6b**. Isovalue set at 0.03 a.u.

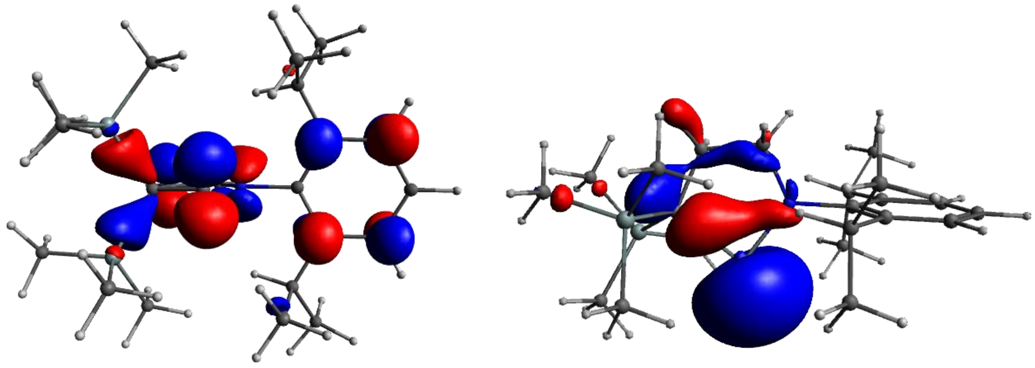

Figure S13. The LUMO (left) and HOMO (right) of **7a**. Isovalue set at 0.045 a.u.

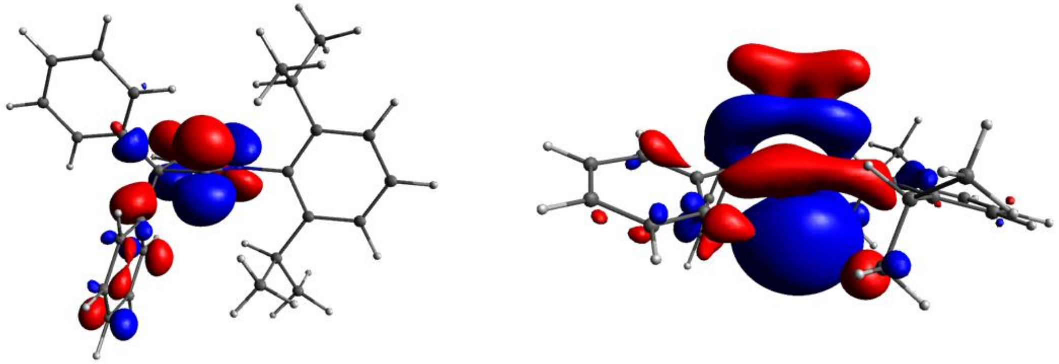

Figure S14. The LUMO (left) and HOMO (right) of **7b**. Isovalue set at 0.05 a.u.

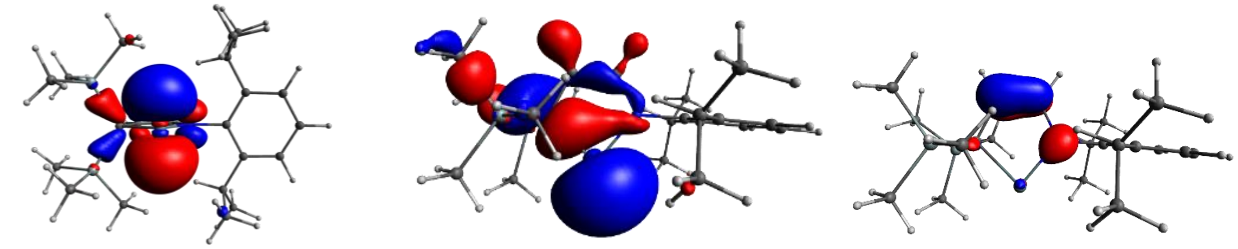

Figure S15. The LUMO (left), HOMO (middle), and HOMO−1 (right) of **8a**. Isovalue set at 0.08 a.u.

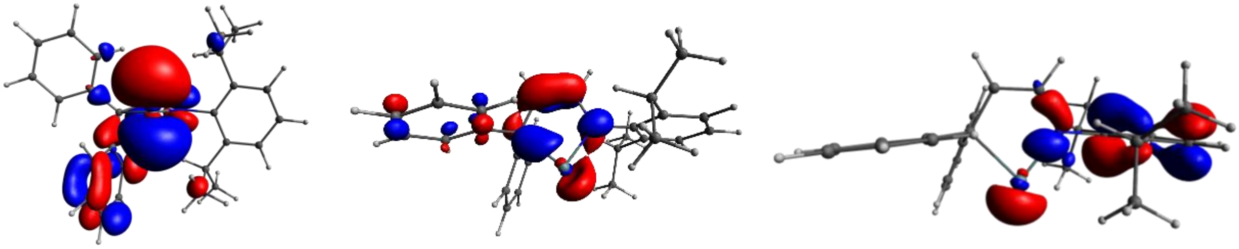

Figure S16. The LUMO, HOMO, and HOMO-2 (from left to right) of **8b**. Isovalue set at 0.06 a.u.

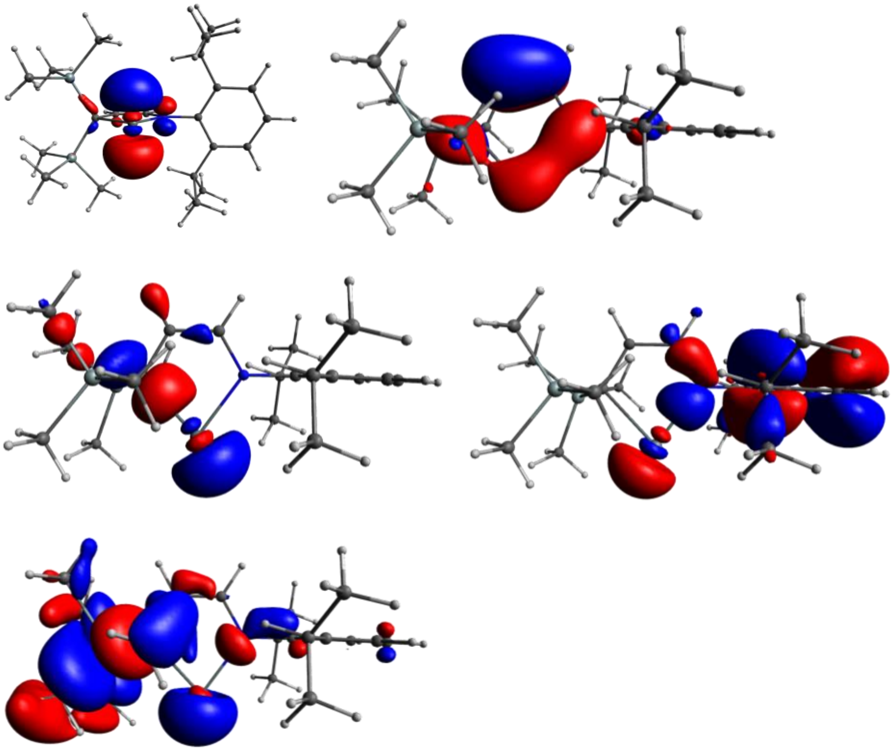

Figure S17. The LUMO (top left), HOMO (top right), HOMO−1 (middle left), HOMO−2 (middle right) and HOMO−5 (bottom) of **9a**. Isovalue set at 0.03 a.u.

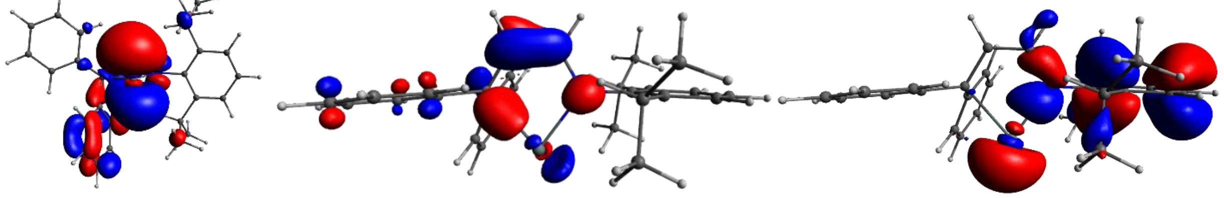

Figure S18. The LUMO (left), HOMO (middle), HOMO−2 (right) of **9b**. Isovalue set at 0.06 a.u.

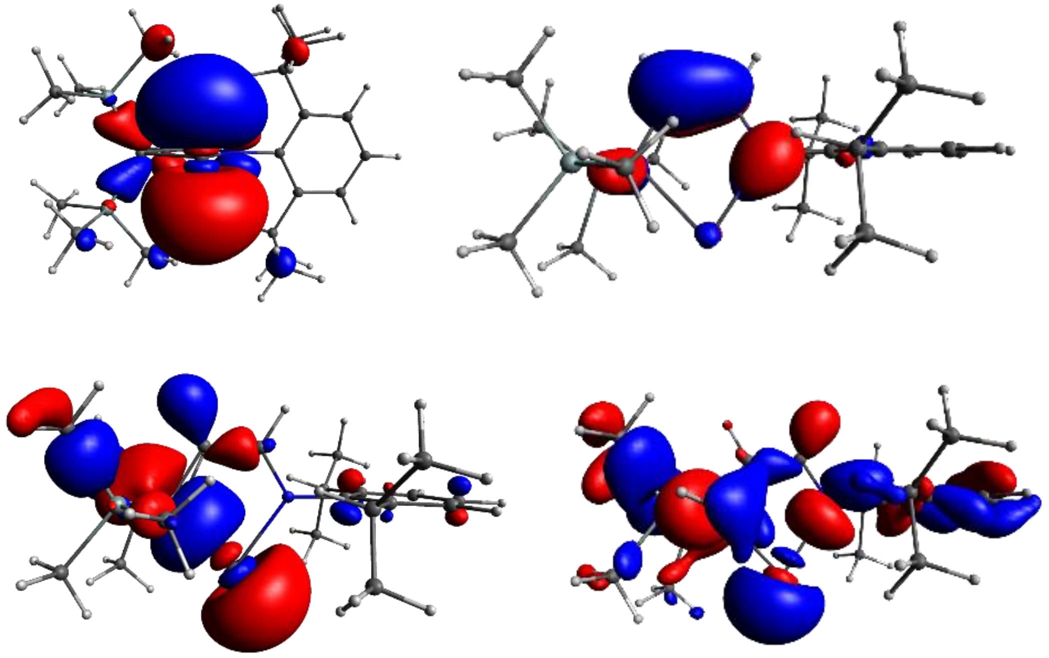

Figure S19. The LUMO (upper left), HOMO (upper right), HOMO-1 (lower left) and HOMO-8 (lower right) of **10a**. Isovalue set at 0.03 a.u.

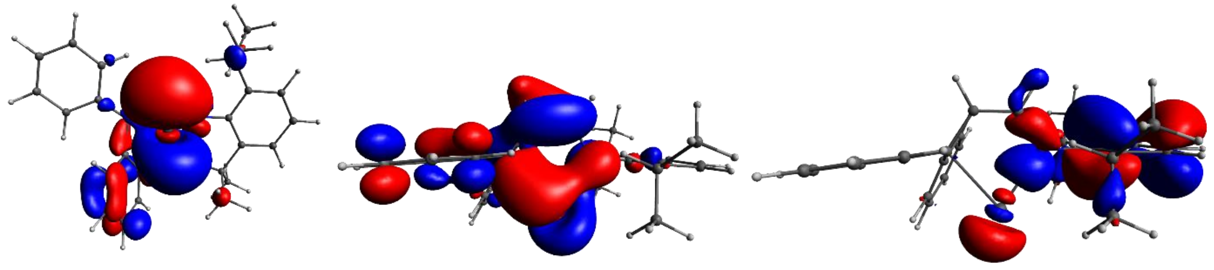

Figure S20. The LUMO, HOMO, and HOMO-2 (from left to right) of **10b**. Isovalue set at 0.03 a.u.

3. EDA-NOCV analysis

Table S2. A summary of the results from EDA-NOCV calculations (kcal mol^{-1}) for the transition state geometries between CAAE **1a-5a** and H_2.

Energy component (kcal mol^{-1})	**1a+H₂**	**2a+H₂**	**3a+H₂**	**4a+H₂**	**5a+H₂**
ΔE_{Pauli}	233.9	313.5	249.6	227.6	232.6
ΔE_{Elstat}[a]	-88.2 (33.2 %)	-104.1 (37.2 %)	-107.7 (41.6 %)	-105.2 (42.8 %)	-117.4 (46.1 %)
ΔE_{Orb}[a]	-174.2 (65.7 %)	-174.5 (62.4 %)	-149.8 (57.9 %)	-139.2 (56.7 %)	-136.2 (53.4 %)
$\Delta E_{Orb(1)}$[b]	-131.0 (75.2 %)	-102.7 (58.9 %)	-109.2 (72.9 %)	-105.3 (75.6 %)	-101.7 (74.7 %)
$\Delta E_{Orb(2)}$[b]	-39.9 (22.9 %)	-62.4 (35.8 %)	-32.9 (22.0 %)	-27.3 (19.6 %)	-27.4 (20.1 %)
ΔE_{Disp}[a]	-2.9 (1.1 %)	-1.1 (0.4 %)	-1.3 (0.5 %)	-1.3 (0.5 %)	-1.3 (0.5 %)
ΔE_{Int}	-31.3	33.8	-9.2	-18.0	-22.2

[a]Percentage contribution from overall attractive forces in brackets.
[b]Percentage contribution from total orbital interaction energy.

Table S3. A summary of the results from EDA-NOCV calculations (kcal mol^{-1}) for the transition state geometries between CAAE **1a-2a** and CO_2.

Energy component (kcal mol^{-1})	**1a+CO₂**	**2a+CO₂**
ΔE_{Pauli}	32.8	145.6
ΔE_{Elstat}[a]	-26.8 (59.6 %)	-80.1 (47.5 %)
ΔE_{Orb}[a]	-12.7 (28.2 %)	-85.2 (50.5 %)
$\Delta E_{Orb(1)}$[b]	-9.4 (74.0 %)	-69.9 (82.0 %)
ΔE_{Disp}[a]	-5.5 (12.2 %)	-3.4 (2.0 %)
ΔE_{Int}	-12.2	-23.2

[a]Percentage contribution from overall attractive forces in brackets.
[b]Percentage contribution from total orbital interaction energy.

a)

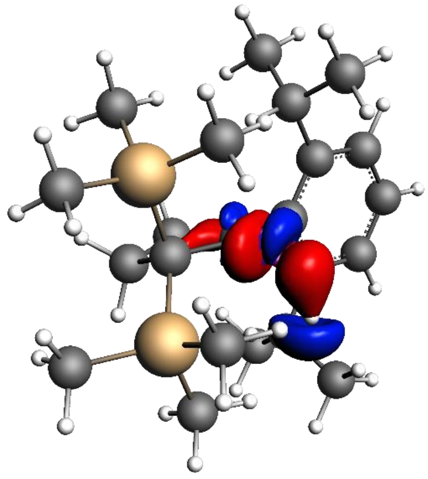

ΔE_1 = -131.0 kcal mol^{-1}; ν = ± 1.00

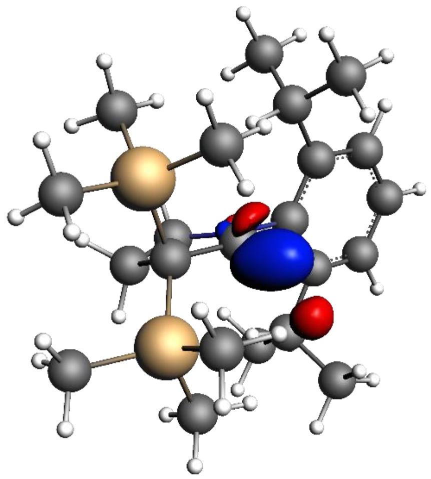

ΔE_2 = -39.9 kcal mol^{-1}; ν = ± 0.47

b)

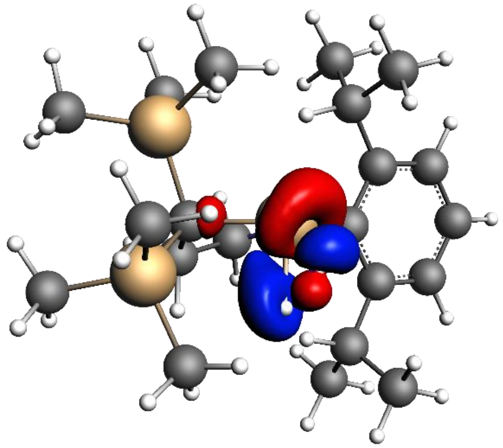

ΔE_1 = -102.7 kcal mol^{-1}; ν = ± 0.88

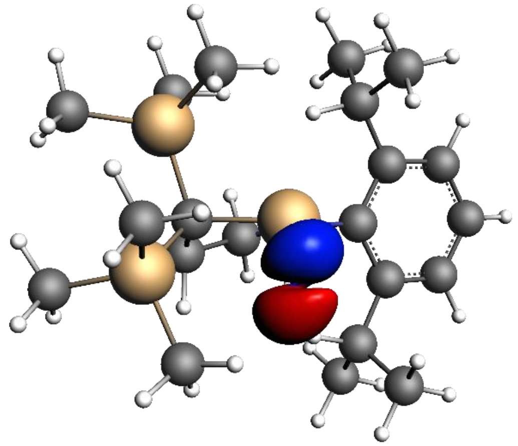

ΔE_2 = -62.4 kcal mol^{-1}; ν = ± 0.55

c)

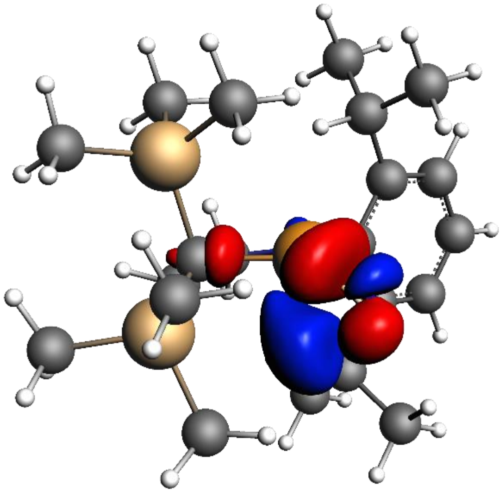

ΔE_1 = -109.2 kcal mol^{-1}; ν = ± 1.16

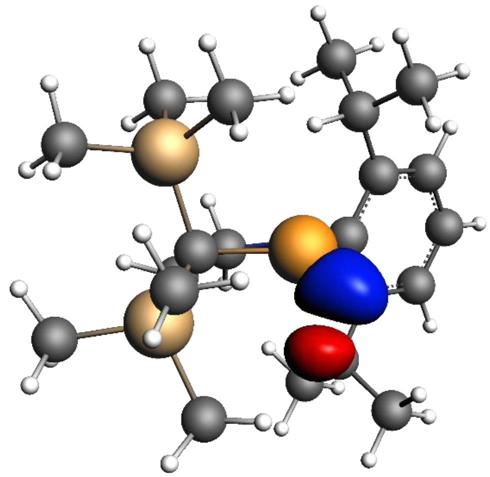

ΔE_2 = -32.9 kcal mol^{-1}; ν = ± 0.48

d)

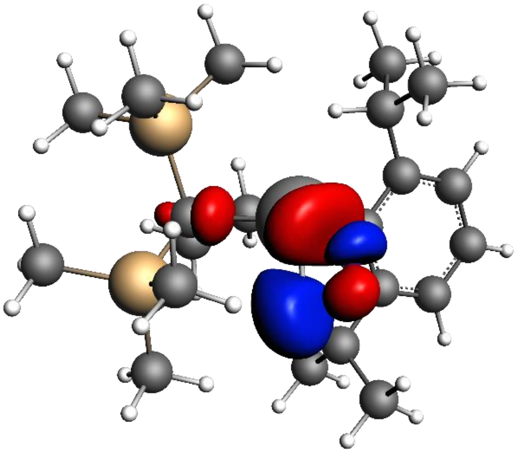

ΔE_1 = -105.3 kcal mol^{-1}; ν = ± 1.24

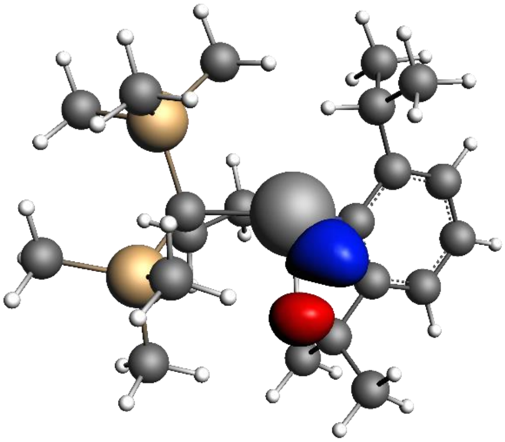

ΔE_2 = -27.3 kcal mol^{-1}; ν = ± 0.48

e)

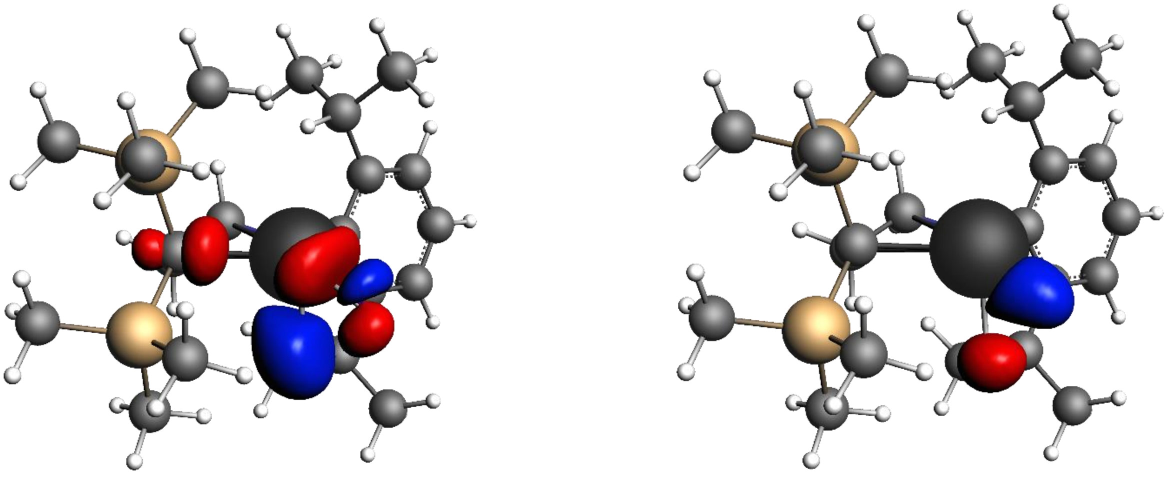

ΔE_1 = -101.7 kcal mol^{-1}; ν = ± 1.28 ΔE_2 = -27.4 kcal mol^{-1}; ν = ± 0.51

Figure S21. Deformation densities $\Delta\rho_1$ ($\Delta E_{Orb(1)}$) and $\Delta\rho_2$ ($\Delta E_{Orb(2)}$) for the transition state geometry of a) **1a**, b) **2a**, c) **3a**, d) **4a** and e) **5a** with H_2. Charge flow is from red to blue.

a) b)

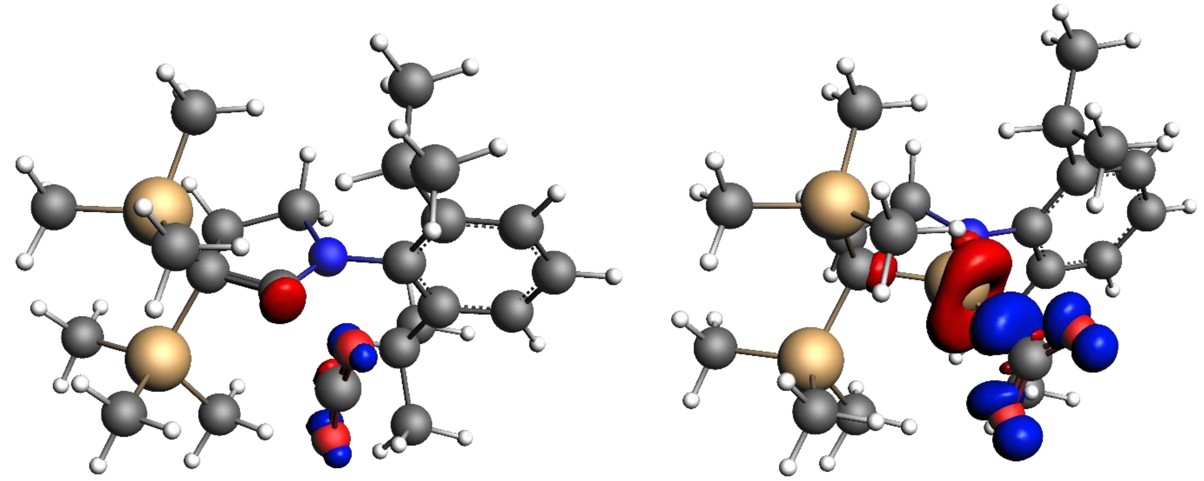

ΔE_1 = -9.4 kcal mol^{-1}; ν = ±0.34 ΔE_2 = -69.9 kcal mol^{-1}; ν = ±0.90

Figure S22. Deformation densities $\Delta\rho_1$ ($\Delta E_{Orb(1)}$) for the transition state geometry of a) **1a+CO_2** and b) **2a+CO_2**. Charge flow is from red to blue.

4. Summary of reaction thermodynamics

Table S4. A summary of the activation energies (kcal mol^{-1}) for oxidative addition between **1-9** and **10b** and H_2.

Compound E = C	ΔG‡	Compound E = Si	ΔG‡	Compound E = Ge	ΔG‡	Compound E = Sn	ΔG‡	Compound E = Pb	ΔG‡
**1a**	24.6	**2a**	40.8	**3a**	50.8	**4a**	58.9	**5a**	72.5
**1b**	28.8	**2b**	37.2	**3b**	49.5	**4b**	61.6	**5b**	73.8
**6a**	26.1	**7a**	40.5	**8a**	50.6	**9a**	61.5	**10a**	52.3*
**6b**	23.9	**7b**	35.9	**8b**	47.9	**9b**	60.6	**10b**	100.4

*Acyclic TS

Table S5. A summary of the Gibbs free energy change (kcal mol^{-1}) for the oxidative addition between **1-10** and H_2.

Compound E = C	ΔG_r	Compound E = Si	ΔG_r	Compound E = Ge	ΔG_r	Compound E = Sn	ΔG_r	Compound E = Pb	ΔG_r
**1a**	−38.2	**2a**	−27.2	**3a**	−8.7	**4a**	7.5	**5a**	32.4
**1b**	−43.0	**2b**	−24.7	**3b**	−5.6	**4b**	11.1	**5b**	35.2
**6a**	−36.6	**7a**	−24.1	**8a**	−5.1	**9a**	11.0	**10a**	34.6* (25.3)
**6b**	−51.0	**7b**	−26.7	**8b**	−8.5	**9b**	9.1	**10b**	33.3

*Cyclic product, (acyclic product)

Table S6. A summary of the activation energies of Werner adduct formation, oxidative addition, and Gibbs free energy change (kcal mol^{-1}) between **1-5** and NH_3 and the optimized E-N bond length (Å) in the CAAE←NH_3 adducts.

Compound	ΔG‡ (Werner adduct)	ΔG‡ (Oxidative addition)	ΔG_r	E-N (adduct)
**1a**	42.3	32.2	−18.3	1.680
**1b**	30.2	51.0	−20.7	1.550
**2a**		36.8	−34.7	2.211
**2b**		35.8	−34.0	2.117
**3a**		49.7	−4.7	2.346
**3b**		50.3	−2.3	2.262
**4a**		58.4	14.6	2.564
**4b**		65.6	17.9	2.479
**5a**		74.4	44.5	2.675
**5b**		74.6	48.5	2.585

Table S7. Summary of the activation energies and Gibbs free energy change in kcal/mol for the activation of CO_2 by **1a-2a** and **6a-7a**.

Compound	$\Delta G^{\ddagger}$	ΔG_r
**1a**	5.4	−13.4
**2a**	16.1	−3.9
**6a**	4.1	−14.1
**7a**	19.3	0.9

5. Transition state structures

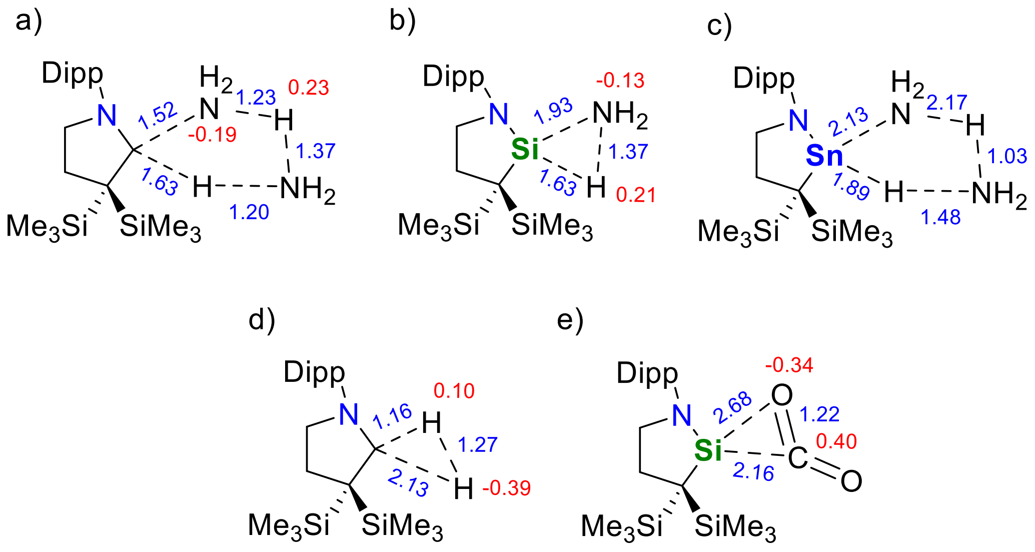

Figure S23. Bond lengths (blue) and Mulliken charges (red) involved in the activation of a) NH_3 by **1a** via proton-shuttling mechanism, b) NH_3 by **2a**, c) NH_3 by **4a** d) H_2 by **1a**, and e) CO_2 by **2a**.

Table S8. Calculated bond lengths (Å), angles (°) and Mulliken charges (q) of the transition state for the activation of H_2 by **1a-10a.**

Compounds	E-H	E-H	H-H	∠H-E-H	q(H1)	q(H2)
**1a**	1.157	2.215	1.269	30.334	-0.3869	0.097784
**2a**	1.679	1.226	1.048	38.443	-0.07164	0.130027
**3a**	1.797	1.551	1.301	45.008	0.170022	-0.0885
**4a**	1.977	1.714	1.458	45.862	0.15157	-0.13819
**5a**	1.755	2.029	1.629	50.348	0.145598	-0.18252
**6a**	1.152	2.128	1.294	31.505	-0.3812	0.109237
**7a**	1.759	1.516	1.197	42.064	0.152081	-0.07308
**8a**	1.829	1.551	1.462	52.863	0.184804	-0.08226
**9a**	1.946	1.150	0.992	22.833	0.158663	-0.13268
**10a**	1.952	1.946	1.193	35.654	-0.06271	-0.0695

Table S9. Calculated bond lengths (Å), angles (°) and Mulliken charges (q) of the transition state for the activation of H_2 by **1b-10b.**

Compounds	E-H	E-H	H-H	∠H-E-H	q(H1)	q(H2)
**1b**	1.246	1.832	0.982	30.243	-0.10901	0.133577
**2b**	1.726	1.516	1.205	43.024	-0.05341	0.137923
**3b**	1.726	1.516	1.205	43.025	-0.06287	0.165795
**4b**	1.951	1.716	1.469	46.692	-0.1176	0.142916
**5b**	2.018	1.769	1.646	51.014	-0.16959	0.121169
**6b**	1.271	1.835	0.951	29.034	-0.07241	0.12407
**7b**	1.515	1.689	1.211	43.994	0.331424	-0.11236
**8b**	1.743	1.554	1.332	47.24	0.174961	-0.01068
**9b**	1.918	1.725	1.494	48.056	0.014082	-0.0806
**10b**	1.941	1.958	1.275	38.185	-0.07432	-0.0944

Table S10. Calculated bond lengths (Å), angles (°) and Mulliken charges (q) of the transition state for the activation of NH_3 by **1a-5a.**

Compounds	E-H	E-N	N-H	∠N-E-H	qN	qH
**1a**	1.115	2.467	1.774	40.442	-0.95562	0.155778
**2a**	1.633	1.931	1.372	44.29	-0.12524	0.21362
**3a**	1.634	2.037	1.499	46.64	-0.66227	0.198839
**4a**	1.784	2.21	1.647	47.22	-0.76604	0.170131
**5a**	1.8	2.302	1.812	50.532	-0.82568	0.154658

Table S11. Calculated bond lengths (Å) and angles (°) of the transition state for the activation of NH_3 by **1a-5a** via proton-shuttling mechanism**.**

Compounds	E-H	E-N	N-H	H-N	N-H	∠N-E-H
**1a**	1.625	1.521	1.228	1.366	1.196	83.784
**2a**	2.065	1.898	1.637	1.102	1.162	77.063
**3a**	3.583	1.989	1.775	1.069	1.019	67.58
**4a**	1.887	2.132	2.166	1.034	1.479	83.84
**5a**	1.812	2.229	2.199	1.03	1.771	86.791

Table S12. Calculated bond lengths (Å) and Mulliken charges (q) of the transition state for the activation of CO_2 by **1a** and **6a.**

Compounds	E-C	qE	qC
**1a**	2.521	-0.54955	0.439577
**6a**	2.575	-0.6011	0.446656

Table S13. Calculated bond lengths (Å) and Mulliken charges (q) of the transition state for the activation of CO_2 by **2a** and **7a.**

Compounds	E-C	C-O	E-O	qE	qC	qO
**2a**	2.155	1.216	2.675	-0.22918	0.3953	-0.34286
**7a**	2.158	1.219	2.493	-0.16523	0.343367	-0.30975

6. Calculated energy diagrams

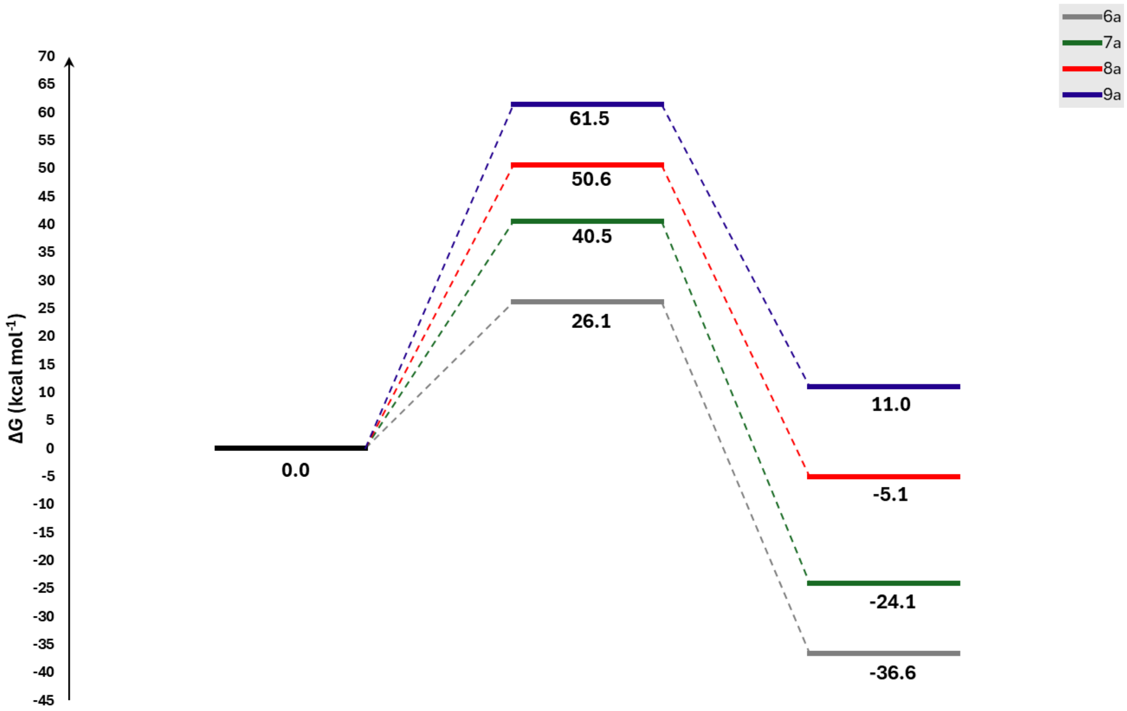

Figure S24. Calculated energy profile for the activation of H_2 by **6a-9a.**

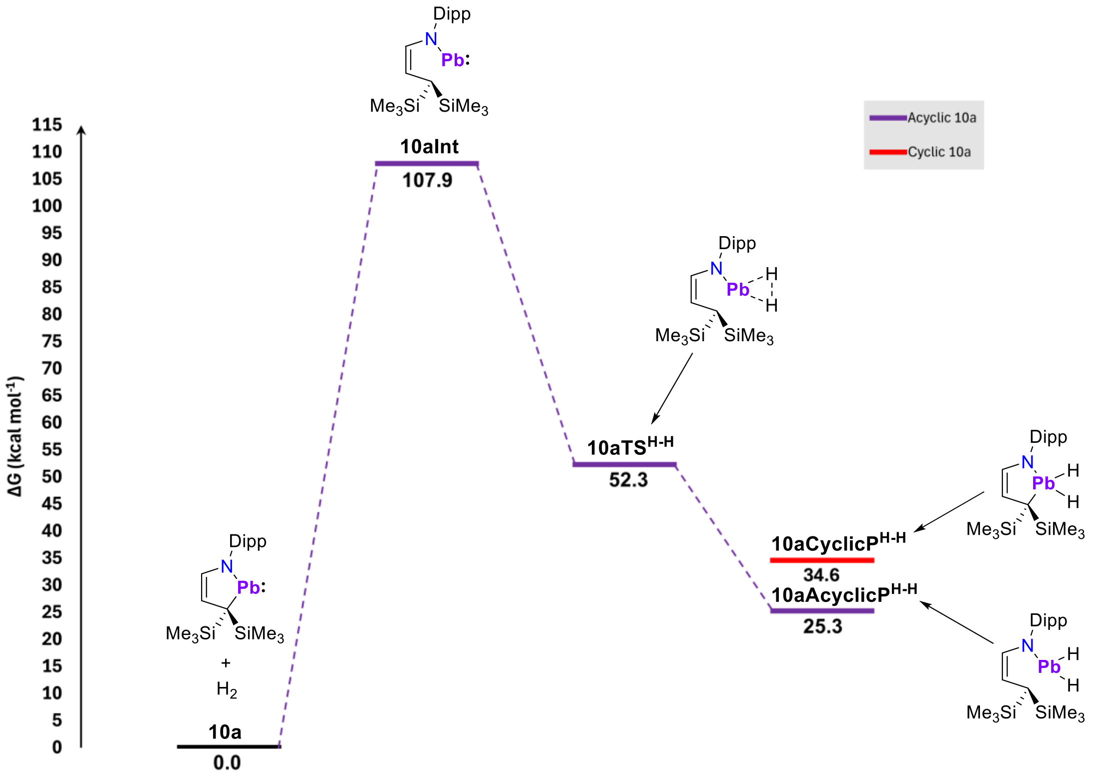

Figure S25. Calculated energy profile diagram for the activation of H_2 by **10a**.

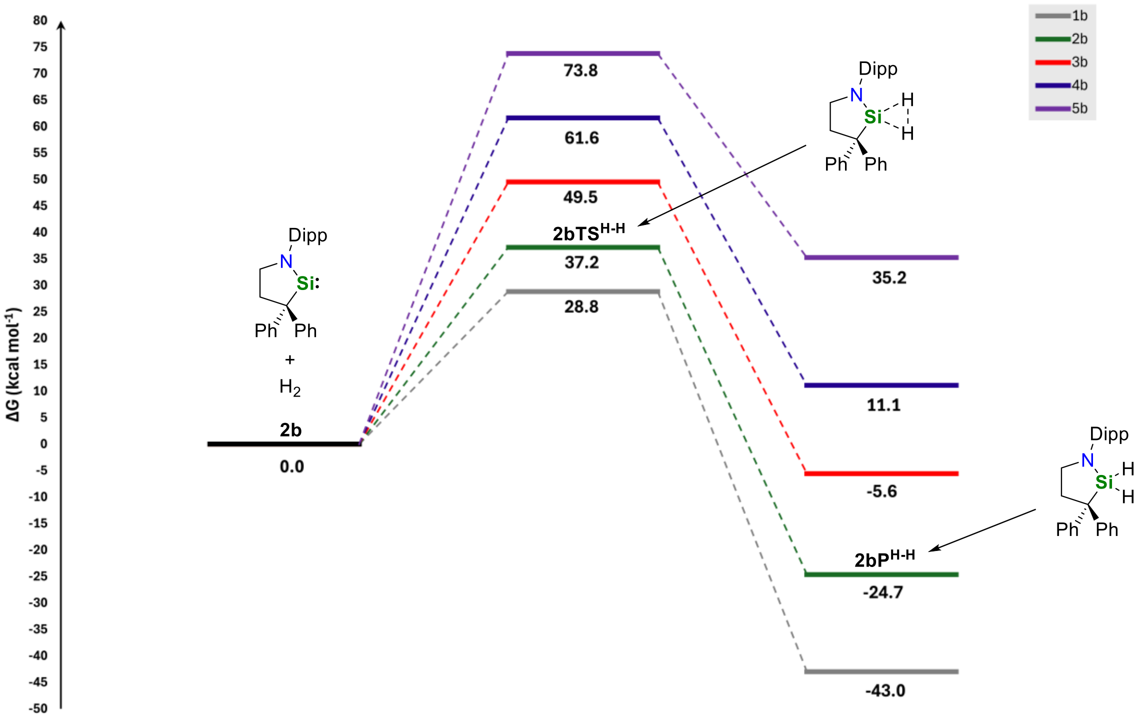

Figure S26. Reaction profile diagram for the activation of H_2 by **1b-5b**.

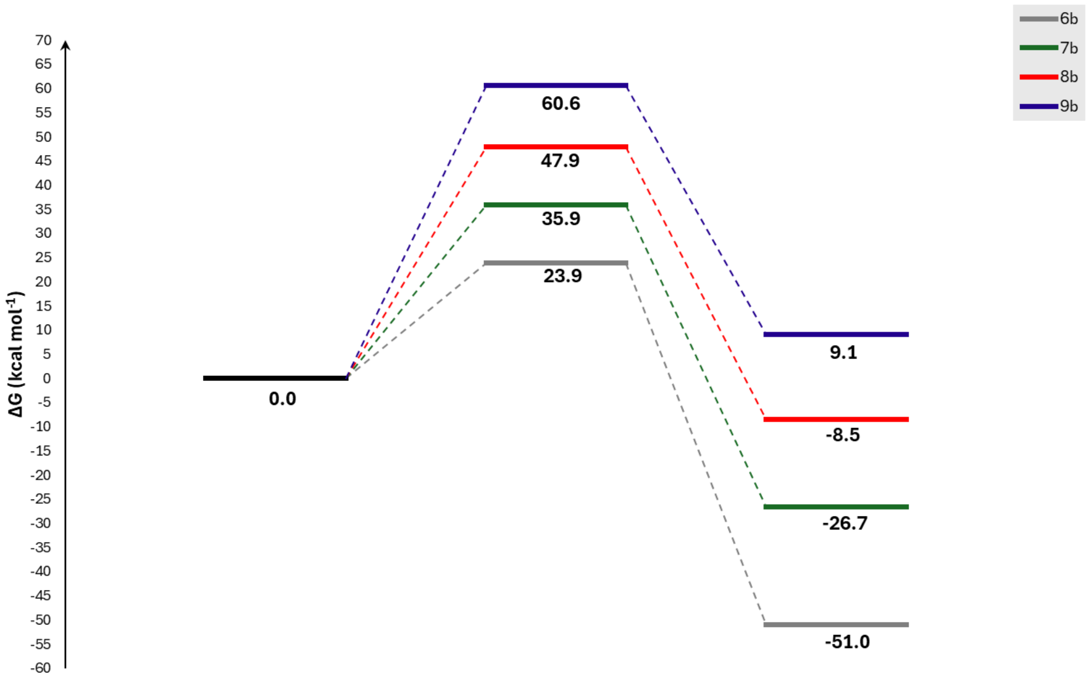

Figure S27. Calculated energy profile for the activation of H_2 by **6b-9b.**

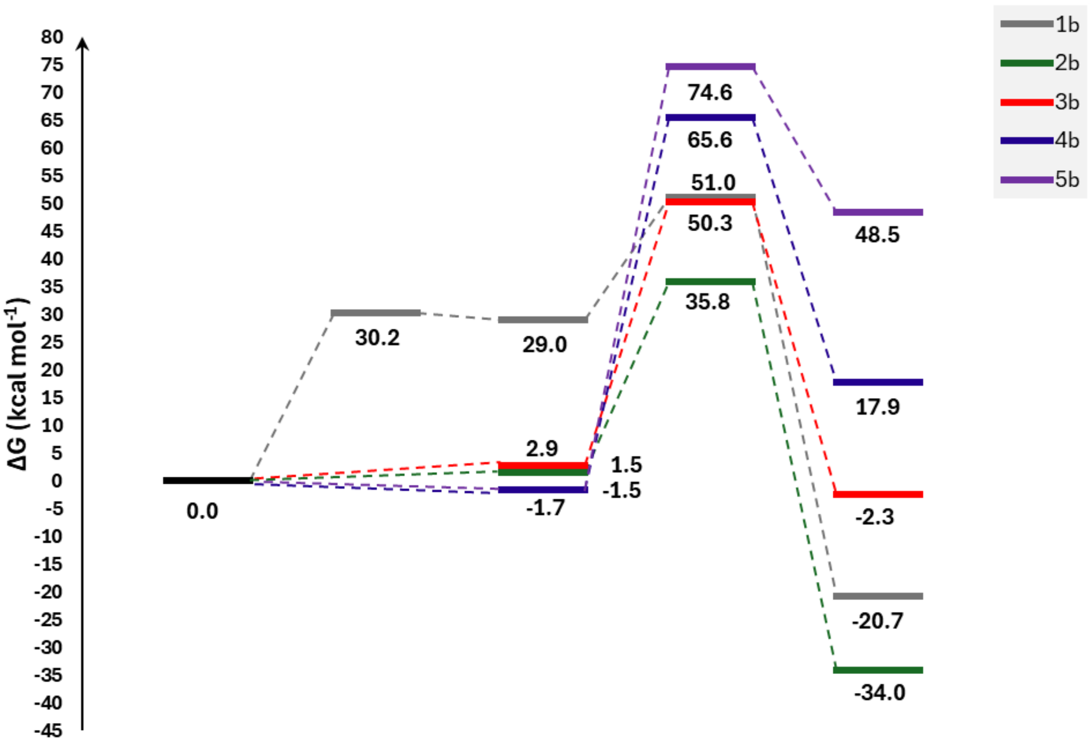

Figure S28. Calculated energy profile for the activation of NH_3 by **1b-5b**.

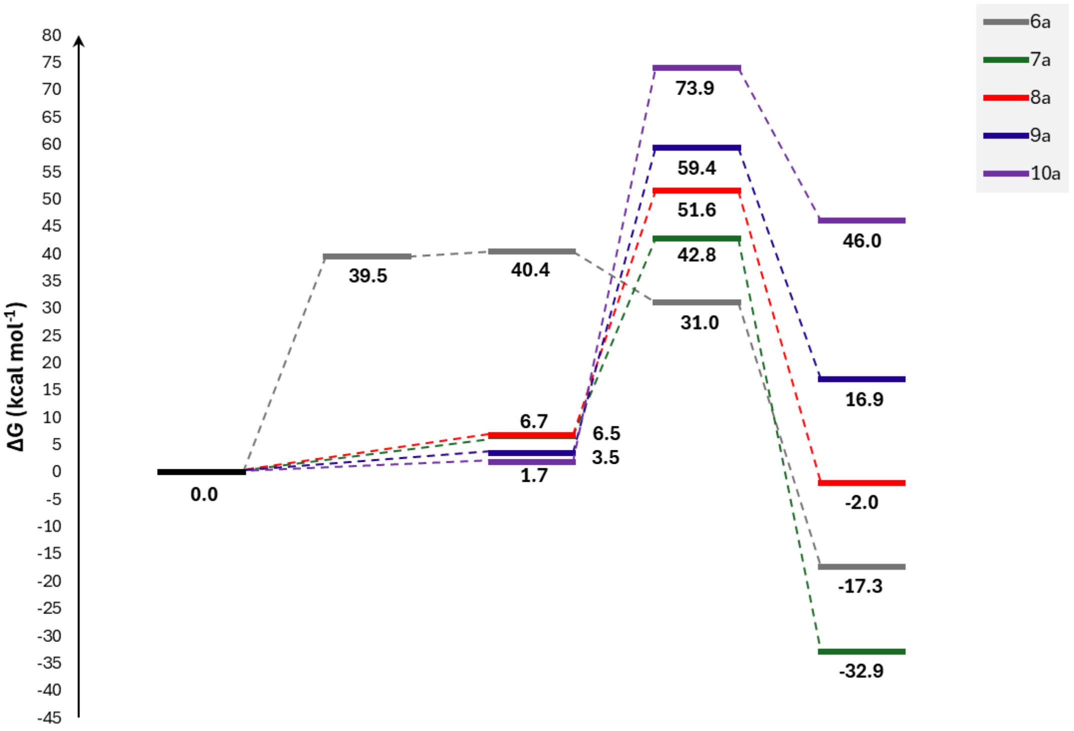

Figure S29. Calculated energy profile for the activation of NH_3 by **6a-10a**.

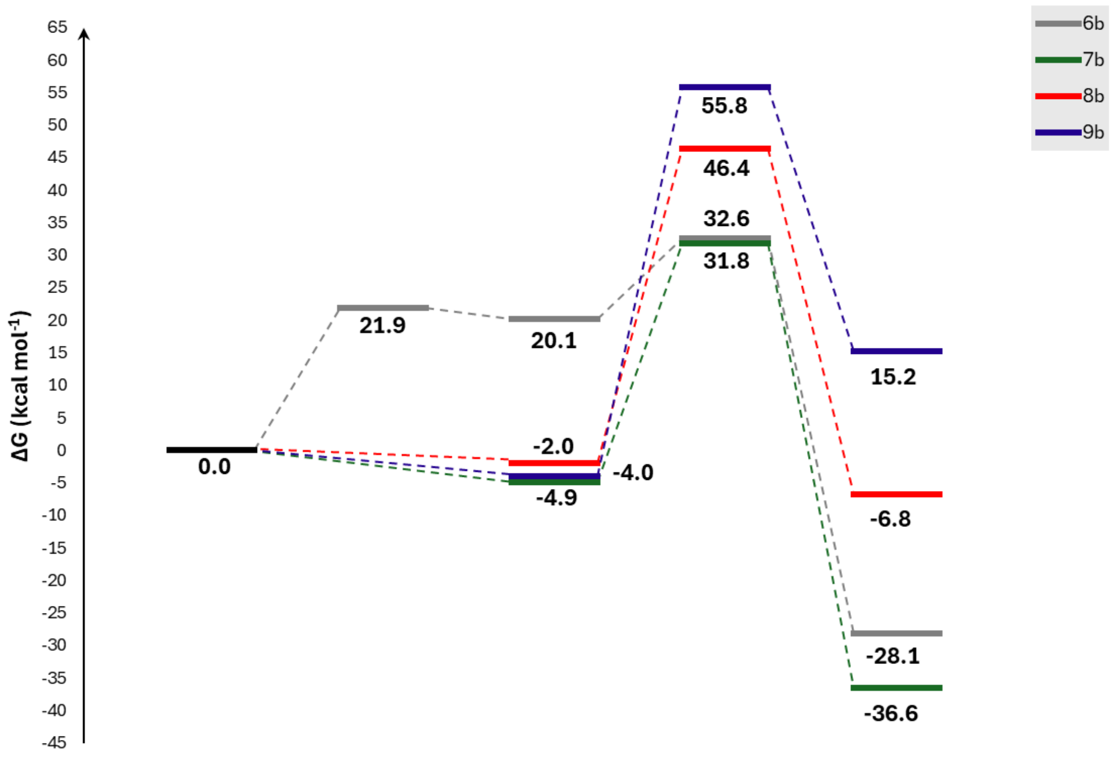

Figure S30. Calculated energy profile for the activation of NH_3 by **6b-10b**.

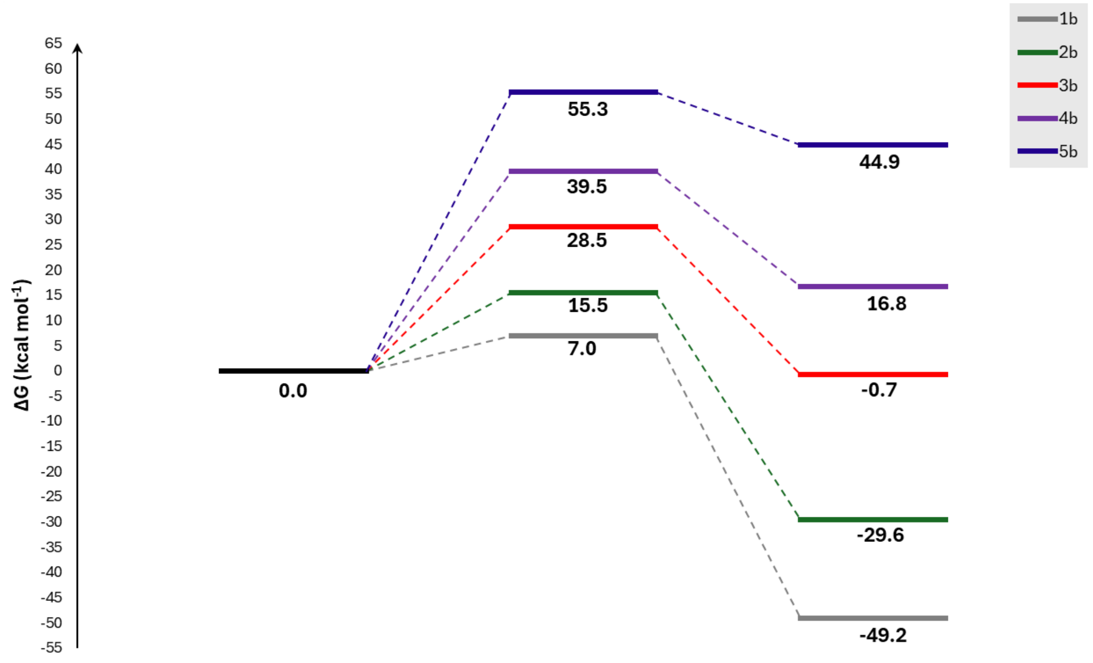

Figure S31. Calculated energy profile for the activation of NH_3 by **1b-5b** via proton-shuttling mechanism.

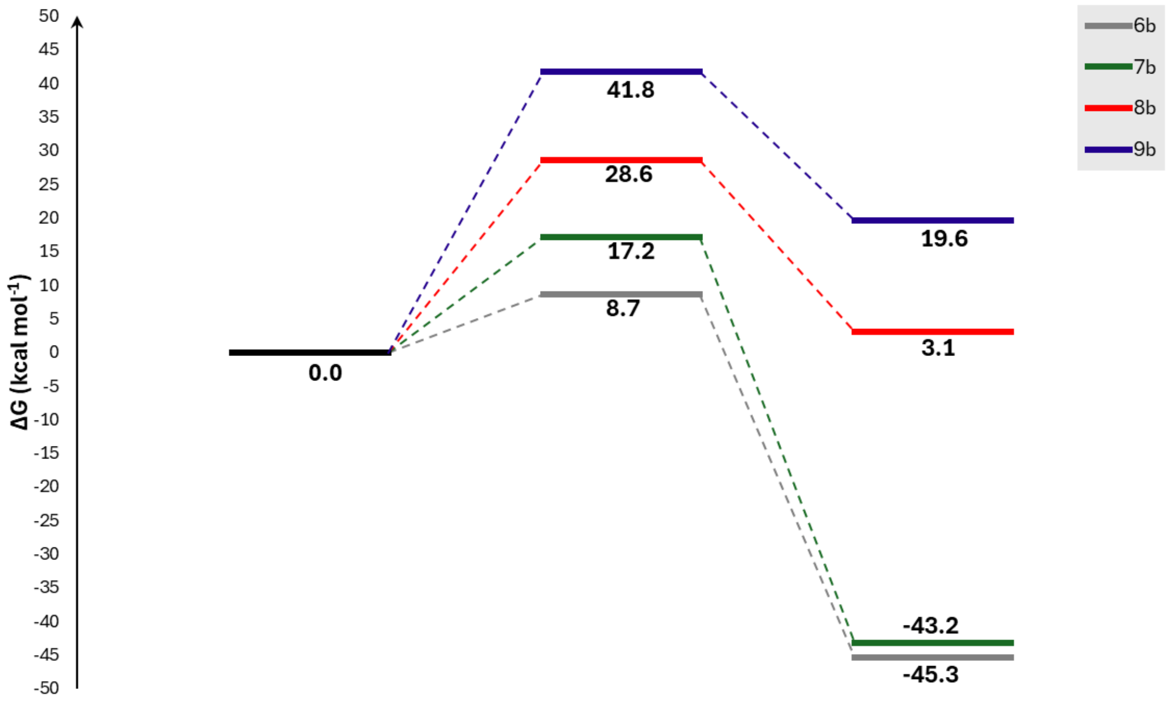

Figure S32. Calculated energy profile for the activation of NH_3 by **6b-10b** via proton-shuttling mechanism.

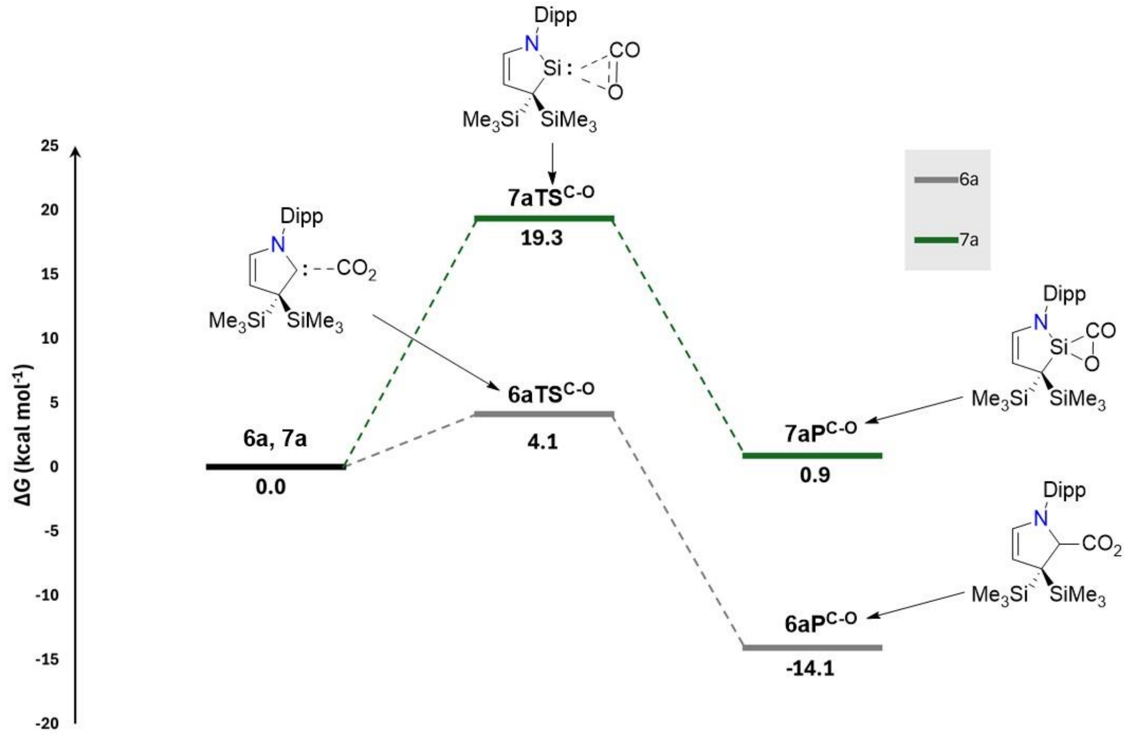

Figure S33. Calculated energy profile for the activation of CO_2 by **6a** and **7a**.

7. References

(1) Turner, Z. R. Chemically Non-Innocent Cyclic (Alkyl)(Amino)Carbenes: Ligand Rearrangement, C−H and C−F Bond Activation. *Chem. – Eur. J.* **2016**, *22* (32), 11461–11468. https://doi.org/10.1002/chem.201602264.

(2) Lundahl, J.; English, L. E.; Ward, J. S.; Vasko, P. Experimental and Computational Studies of Amido Chlorogermylenes as Lewis Acids in CO2 Insertion. *Eur. J. Inorg. Chem.* **2025**, *28* (14), e202500116. https://doi.org/10.1002/ejic.202500116.

(3) Kosai, T.; Ishida, S.; Iwamoto, T. A Two-Coordinate Cyclic (Alkyl)(Amino)Silylene: Balancing Thermal Stability and Reactivity. *Angew. Chem. Int. Ed.* **2016**, *55* (50), 15554–15558. https://doi.org/10.1002/anie.201608736.

(4) Wang, L.; Lim, Y. S.; Li, Y.; Ganguly, R.; Kinjo, R. Isolation of a Cyclic (Alkyl)(Amino)Germylene. *Molecules* **2016**, *21* (8), 990. https://doi.org/10.3390/molecules21080990.

(5) Ghosh, B.; Bharadwaz, P.; Sarkar, N.; Phukan, A. K. Activation of Small Molecules by Cyclic Alkyl Amino Silylenes (CAASis) and Germylenes (CAAGes): A Theoretical Study. *Dalton Trans.* **2020**, *49* (39), 13760–13772. https://doi.org/10.1039/D0DT03043K.
